# Heterostructured Materials for Room‐Temperature Na‐S Batteries: Directional Design Strategies and Multifaceted Applications

**DOI:** 10.1002/advs.76019

**Published:** 2026-08-06

**Authors:** Yeqing Yang, Xue Li, Kunjie Zhu, Xiang‐Long Huang, Yun‐Xiao Wang

**Affiliations:** ^1^ Institute of Energy Materials Science University of Shanghai for Science and Technology Shanghai P. R. China; ^2^ Guangdong Provincial Key Laboratory of New Energy Materials Service Safety Shenzhen Key Laboratory of Polymer Science and Technology College of Materials Science and Engineering Shenzhen University Shenzhen P. R. China; ^3^ Australian Institute of Innovative Materials University of Wollongong, Innovation Campus University of Wollongong Wollongong Australia

**Keywords:** carbons, heterostructure, Na metal anode, RT Na‐S batteries, solid‐state electrolyte

## Abstract

The development of room‐temperature sodium‐sulfur (RT Na‐S) batteries is fundamentally constrained by many severe challenges, including the polysulfide shuttle effect, sluggish sulfur conversion kinetics, and unstable sodium metal anode. This comprehensive review systematically presents a universal and versatile materials design methodology, heterostructure engineering, to concurrently address these interlinked challenges. The electrochemical mechanisms of RT Na‐S batteries based on different redox pathways are elaborated, with their corresponding key challenges. Followed by that, the multifunctional advantages and fundamental design principles of heterostructured materials are summarized. It is of greater importance that we pioneer to comprehensively elucidate various real applications of these tailored heterostructured materials in advanced RT Na‐S batteries, involving host materials for sulfur and sodium, interface layer materials for sulfur cathode and sodium anode, solid‐state electrolytes, and current collectors, etc. We conclude with critical perspectives on the unresolved scientific questions and practical hurdles, charting a pathway from laboratory‐scale innovation to the realization of high‐energy, long‐cycle‐life, and safe devices. By demonstrating how tailored heterointerfaces can regulate sulfur speciation, guide sodium electrodeposition, and accelerate reaction kinetics, this review establishes heterostructure engineering as a cornerstone for advancing RT Na‐S batteries.

## Graphical abstract

In this work, heterostructured materials for room‐temperature Na‐S batteries are comprehensively reviewed. The electrochemical mechanisms of Na‐S batteries based on various redox pathways are clarified. The intrinsic functionalities and design principles of heterostructured materials are summarized. The emphasis is on their multifaceted applications, including hosts for sodium anode and sulfur cathode, interface layers for electrodes, solid‐state electrolytes, and current collectors.

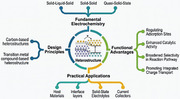

## 1 Introduction

The global transition toward renewable energy and sustainable electrification has created an urgent demand for energy storage systems that are cost‐effective, resource‐abundant, and capable of high energy density [1, 2, 3]. Room‐temperature sodium‐sulfur (RT Na‐S) batteries stand out as one of the prime candidates to meet this demand, primarily due to the compelling advantages in key materials. Sodium is one of the most abundant elements on the Earth's crust, ensuring low cost and eliminating the geopolitical concerns associated with lithium and cobalt [4, 5]. Sulfur, a byproduct of industrial processes, is similarly inexpensive and offers a high theoretical specific capacity of 1675 mA h g^−1^. The coupling of a sodium anode (theoretical capacity: 1166 mA h g^−1^) with a sulfur cathode enables a high theoretical energy density, positioning RT Na‐S batteries as a viable technology for large‐scale grid storage and beyond [6, 7, 8].

Despite this promising outlook, the practical realization of high‐performance RT Na‐S batteries is severely hampered by a complex set of intertwined electrochemical challenges rooted in the intrinsic properties of sulfur and sodium. These challenges manifest separately at the cathode and anode, yet are destructively linked during operation. At the sulfur cathode, the insulating nature of both elemental sulfur (S_8_) and its discharge products (Na_2_S_2_/Na_2_S) necessitates sophisticated conductive scaffolds to assist in charge transport [9, 10, 11]. More critically, the intermediate species generated during cycling, sodium polysulfides (Na_2_S_x_, 4 ≤ x ≤ 8), migrate freely between electrodes under an ether‐based electrolyte system (i.e., the shuttle effect) or undergo nucleophilic reactions with carbonate ester solvents [12, 13]. This leads to irreversible active material loss, rapid capacity fading, and low Coulombic efficiency. Furthermore, the conversion reactions between sulfur and Na_2_S are kinetically sluggish, resulting in high polarization, poor rate capability, and incomplete sulfur utilization [14, 15]. At the sodium metal anode, uncontrolled dendrite growth poses serious safety risks from internal short circuits. This is compounded by a highly reactive and unstable interface with the electrolyte, leading to continuous consumption of both sodium and electrolyte to form a fragile solid‐electrolyte interphase (SEI) [16, 17]. Critically, the polysulfides from the cathode chemically corrode the sodium anode, further destabilizing the SEI and accelerating cell failure. Thus, the problems of the sulfur cathode and sodium anode are not isolated, and they create a vicious cycle that has been the central bottleneck for the development of RT Na‐S batteries.

To address these challenges, conventional materials engineering has largely followed an approach to target a single problem, which has proven insufficient. For the sulfur hosts, conductive carbon matrices (e.g., porous carbons) improve the electronic conductivity of cathodes but only exhibit a weak physical confinement effect for polysulfides via van der Waals forces, failing to suppress long‐term polysulfide shuttling behaviors [18, 19]. Conversely, polar materials like metal oxides offer strong chemical adsorption to anchor polysulfides but often suffer from poor intrinsic electronic conductivity and greatly limited catalytic activity for polysulfide conversion, posing the formation of inactive dead sulfur [20, 21, 22]. For the sodium anode, strategies such as using porous current collectors or applying single‐component artificial SEI layers can partially improve plating uniformity and/or interface stability [23, 24]. However, they typically address only one aspect (e.g., mechanical blocking or ionic conductivity) and are often incapable of simultaneously regulating Na^+^ flux, suppressing dendrites, stabilizing the SEI, and resisting polysulfide corrosion. As a matter of fact, the fundamental limitation of conventional material systems lies in the difficulty of integrating multiple essential functions, including confinement, conductivity, catalysis, and stability, into a single and homogeneous material phase. This functional incompatibility of single‐component systems underscores the shift of the material design concept toward more sophisticated and multifunctional material architectures.

Heterostructure materials, engineered by intimately coupling two or more distinct nanoscale phases (e.g., metal/compound or compound/carbon), have emerged as a transformative design approach that is expected to overcome the abovementioned limitations [25, 26, 27]. Their key feature resides in the synergistic effects generated at the well‐defined internal interfaces, which can become electrochemically active centers for related reactions. The core multifunctional advantages stem from two key interfacial phenomena. First, the difference in Fermi levels between the coupled phases drives electron transfer across the interface, creating a built‐in electric field [28]. This effect acts as a powerful internal pump that drastically enhances charge transfer kinetics and can guide ion flux, thereby accelerating redox reactions and lowering polarization. Second, the different chemical properties of the constituent phases enable synergistic functionality to enable multifunctionality for RT Na‐S batteries [29]. For instance, in a heterostructure of a polar metal sulfide (strong polysulfide adsorbent) and a conductive metal nitride (good electron conductor), their phase interface can not only trap polysulfides via strong chemisorption but also rapidly deliver electrons to catalyze their conversion, effectively transforming a trapping site into a reaction site. This principle can be extended to the anode side, where heterostructures can be designed to combine a phase that promotes Na^+^ diffusion with another that provides mechanical strength, thereby guiding uniform plating and suppressing dendrites [30, 31, 32]. By rationally selecting and integrating different phases, heterostructure materials can be tailored to concurrently fulfill the roles of conductor, adsorbent, catalyst, and stabilizer, which are often mutually exclusive in single‐phase materials.

**FIGURE 1:**
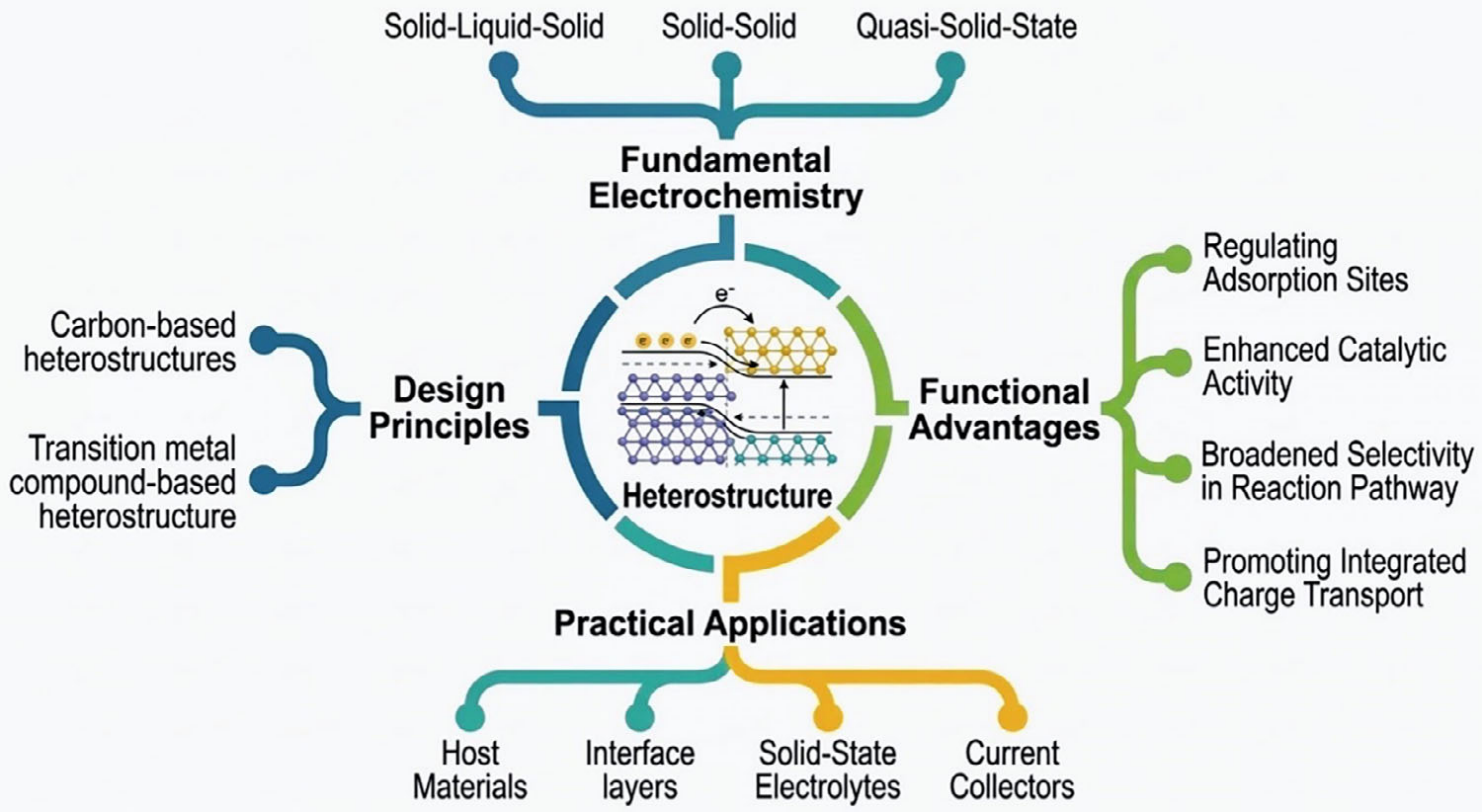
The overview of this review.

Given the aforesaid considerations, this review aims to provide a comprehensive and in‐depth analysis of the rational design, multifunctional mechanisms, and broad applications of heterostructure materials across the entire architecture of RT Na‐S batteries (Figure 1). We move beyond the common narrative focused solely on sulfur cathodes to present a holistic and cell‐level engineering perspective. First, we discuss the mainstream redox mechanisms in RT Na‐S batteries and point out their corresponding key challenges. The functional advantages and design principles of heterostructure materials are stated, detailing the physics of the built‐in electric field, the chemistry of synergistic adsorption/catalysis, and guidelines for phase selection and interface engineering. Subsequently, the central part is organized by critical cell components, systematically examining how heterostructure engineering tackles specific challenges in each domain: (1) advanced sulfur hosts for polysulfide confinement and conversion catalysis; (2) modified sodium hosts or interfacial layers for dendrite suppression and anode stabilization; (3) functional interlayers acting as polysulfide barriers and redox mediators; (4) solid‐state electrolyte to realize superior ion conduction and stable interfaces, and (5) current collectors to regulate sodium deposition and sulfur conversion. Finally, a perspective is provided to clarify the remaining challenges and guide future research directions in RT Na‐S batteries. Through this systematic understanding, this review underscores heterostructure materials as a pivotal key to unlocking high‐energy Na‐S battery technology.

## 2 Fundamental Electrochemistry and Scientific Challenges

**FIGURE 2:**
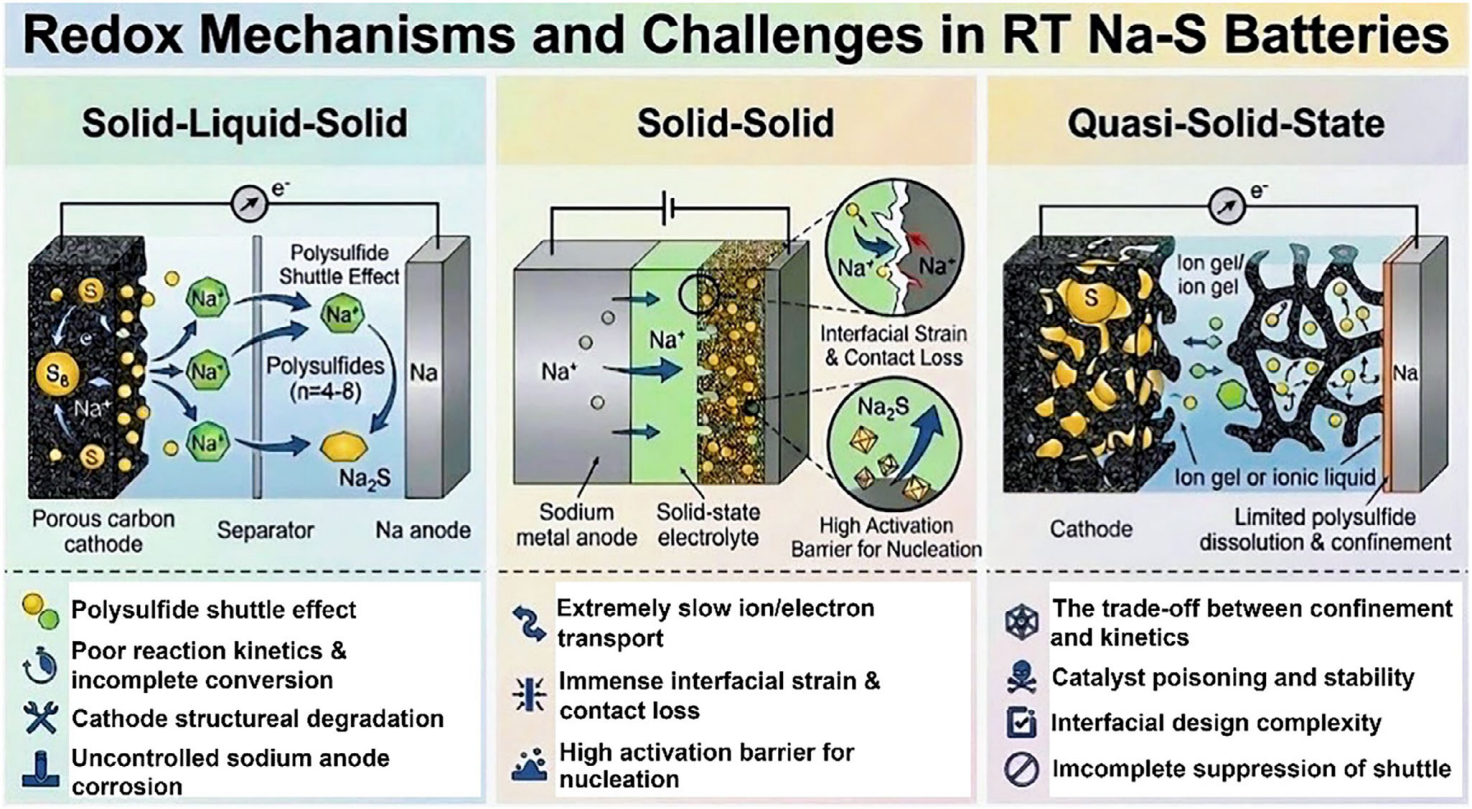
Schematic illustrations of various redox mechanisms in RT Na‐S batteries and corresponding challenges.

The performance of RT Na‐S batteries is fundamentally governed by the intricate phase transformation pathways of sulfur during the discharge–charge process. These pathways, which dictate reaction kinetics, reversibility, and failure modes, can be classified into three archetypes based on their reaction characteristics, as illustrated in Figure 2. A deep understanding of their distinct mechanisms will be essential for rational material design.

### 2.1 Solid‐Liquid‐Solid Reaction

This conventional pathway, prevalent in ether‐based electrolytes, involves a sequential phase evolution: solid S_8_ → soluble long‐chain Na_2_S_n_ (liquid) → solid short‐chain Na_2_S_2_/Na_2_S [33, 34]. The reduction commences with the electrochemical cleavage of the S_8_ ring, forming soluble high‐order polysulfides (Na_2_S_6‐8_). This is followed by a series of stepwise reduction and disproportionation reactions in the electrolyte phase, progressively shortening the polysulfide chains. The defining dynamic of this pathway is the continuous dissolution‐precipitation equilibrium. Soluble intermediates can diffuse freely, creating a homogeneous but problematic liquid‐phase reaction medium. The final rate‐limiting step is the heterogeneous nucleation and growth of insulating Na_2_S_2_ and Na_2_S onto conductive surfaces from the saturated polysulfide solution [35, 36]. This process is inherently slow and sensitive to the local supersaturation and available nucleation sites, often leading to incomplete precipitation as well as large and passivating solid agglomerates. The corresponding voltage profile typically exhibits two distinct plateaus: a higher plateau corresponds to the liquid‐phase reduction to Na_2_S_4_, and a lower and more sloping plateau is associated with the sluggish solid‐phase formation.

#### 2.1.1 Core Scientific Challenges

##### 2.1.1.1 The Shuttle Effect of Polysulfide

The dissolution of long‐chain Na_2_S_n_ is the root cause of the infamous shuttle phenomenon. These soluble species diffuse freely between the cathode and anode. At the sodium metal anode, they are reduced to lower sulfides (chemical discharge), which then diffuse back and are re‐oxidized at the cathode. This parasitic cycle leads to irreversible active material loss, low Coulombic efficiency, rapid capacity fade, and severe self‐discharge behaviors [37, 38].

##### 2.1.1.2 Poor Reaction Kinetics & Incomplete Conversion

The final reduction step from soluble Na_2_S_4_ to insoluble Na_2_S_2_/Na_2_S is kinetically sluggish. The nucleation and growth of these insulating solid products pose a high energy barrier, often posing incomplete conversion, low sulfur utilization, and high polarization [39].

##### 2.1.1.3 Cathode Structural Degradation

The repeated dissolution and precipitation of active material during cycling cause significant volumetric stress and morphological reconstruction of cathode architectures [40, 41]. This easily causes the detachment of active material from the conductive matrix, loss of electrical contact, and eventual electrode pulverization.

##### 2.1.1.4 Uncontrolled Sodium Anode Corrosion

The shuttling polysulfides chemically corrode the sodium metal anode, forming a thick, inhomogeneous, and unstable SEI. This accelerates sodium consumption, promotes dendritic growth, and gives rise to severe safety risks.

### 2.2 Solid‐Solid Reaction

In this desired yet kinetically challenging pathway, sulfur is reduced directly to the discharge products without leaving the solid phase: solid S_8_ → solid Na_2_S_2_/Na_2_S. This is enforced in systems where polysulfide solubility is thermodynamically suppressed, such as in certain solid‐state electrolytes or within ultra‐microporous hosts (<0.5 nm) [42, 43]. The transformation is characterized by a sharp, two‐phase reaction front that propagates through the sulfur particle. The reaction mechanism is dominated by solid‐state ion‐electron transfer across the ever‐growing product layer (Na_2_S). This layer acts as a formidable barrier to both Na^+^ diffusion and electron tunneling, leading to severe polarization and limiting the practical depth of discharge. The nucleation of Na_2_S phase from S_8_ requires overcoming a significant interfacial energy barrier, often resulting in a large voltage hysteresis. The voltage profile for an ideal solid‐solid pathway would theoretically show a single and flat plateau; however, it is actually marked by a single, highly polarized, and sloping plateau, due to the immense interfacial resistance and progressive charge‐transfer limitations.

#### 2.2.1 Core Scientific Challenges

##### 2.2.1.1 Extremely Slow Ion/Electron Transport

In the absence of a liquid medium, the reaction is limited to solid‐solid interfaces. The sluggish solid‐state diffusion kinetics of both Na^+^ ions and electrons through the insulating sulfur and Na_2_S layers result in very high polarization, low practical capacity, and poor rate performance [4, 44].

##### 2.2.1.2 Immense Interfacial Strain & Contact Loss

The direct conversion from S_8_ to Na_2_S theoretically involves a dramatic volumetric expansion of ∼170%. Without a liquid phase to buffer this change, the massive mechanical stress at the rigid solid‐solid interface causes severe contact loss between active material, conductor, and electrolyte, leading to rapid performance degradation.

##### 2.2.1.3 High Activation Barrier for Nucleation

The direct crystallization of Na_2_S from solid S_8_ requires overcoming a formidable nucleation barrier. This often results in significant voltage hysteresis and needs sophisticated cathode architectures with intimate multiphase contact and built‐in catalytic sites to be viable [45, 46].

### 2.3 Quasi‐Solid‐State Reaction

This emerging pathway represents a sophisticated hybrid, where polysulfides are generated but not released into the bulk electrolyte. The sequence can be described as: solid S_8_ → confined/adsorbed Na_2_S_n_ → solid Na_2_S. Here, polysulfide intermediates are chemically tethered to host surfaces or physically confined within nanoscaled pores/matrices [47, 48, 49, 50]. The transformation dynamics shifts from a bulk electrolyte process to heterogeneous reactions occurring within localized high‐concentration microenvironments. The reaction progression is governed by the surface diffusion of adsorbed species and interface‐mediated electron transfer, rather than by long‐range liquid‐phase diffusion. This confinement dramatically alters the reaction coordinates, which can stabilize unusual intermediates, lower local conversion overpotentials through enhanced adsorption, and promote smoother nucleation of the final solids. This results from the pre‐organized and high‐density reactive interfaces. The voltage profile often manifests as a single and consolidated plateau with reduced polarization, compared to the solid‐solid pathway, as the localized liquid‐like environment alleviates some ion transport barriers while effectively suppresses macroscopic polysulfide shuttling.

#### 2.3.1 Core Scientific Challenges

##### 2.3.1.1 The Trade‐Off Between Confinement and Kinetics

Effective physical or chemical confinement often relies on dense or narrow porous structures, which can simultaneously impede electrolyte infiltration and Na^+^ transport [51, 52]. Designing a host that strongly confines polysulfides while maintaining rapid ion access is a critical materials engineering puzzle.

##### 2.3.1.2 Catalyst Poisoning and Stability

Catalysts are essential to accelerate the conversion of confined polysulfides. However, in such a concentrated localized reaction environment, catalyst surfaces are more susceptible to passivation by irreversible sulfur species or degradation products. Maintaining long‐term catalytic activity is a significant challenge to such unique reaction mechanisms.

##### 2.3.1.3 Interfacial Design Complexity

Achieving this pathway requires the precise integration of multiple functions within the cathode: confinement (micro/nanopores, strong adsorption), conduction (electronic and ionic), and catalysis. The rational design and scalable synthesis of such multifunctional hierarchical architectures remain a major hurdle.

##### 2.3.1.4 Incomplete Suppression of Shuttle Behaviors

Under practical conditions (high sulfur loading, lean electrolyte, and/or long cycling), some polysulfide escape is inevitable. The quasi‐solid‐state approach must be coupled with effective barriers or optimized electrolytes to fully suppress long‐range shuttle behaviors.

## 3 Functional Advantages of Heterostructured Materials

**FIGURE 3:**
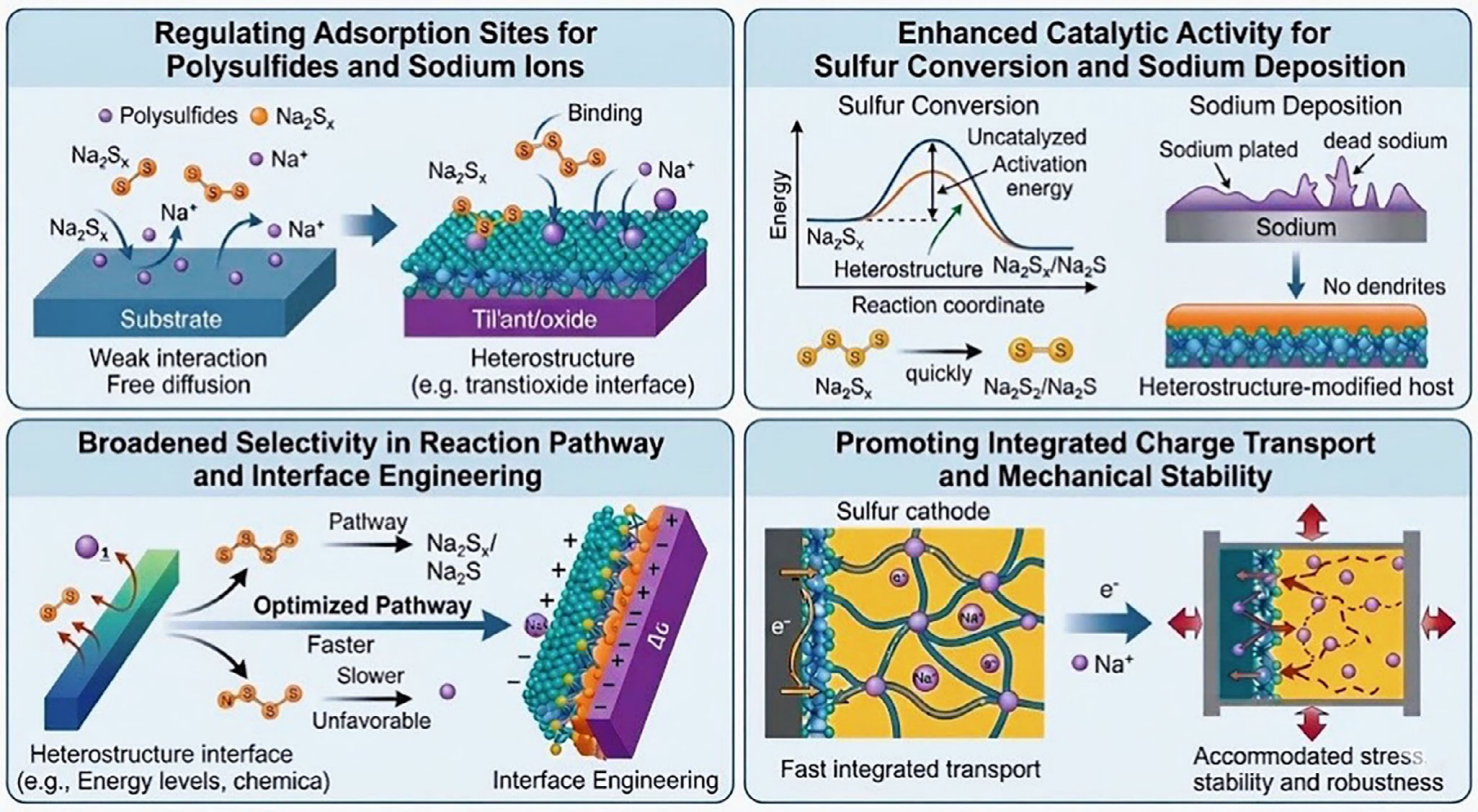
Multifunctionalities of heterostructure materials for RT Na‐S batteries.

Heterostructured materials, through the rational integration of two or more distinct phases, create multifunctional interfaces in the whole material system that uniquely address the interconnected challenges at both the sulfur cathode and sodium metal anode in RT Na‐S batteries. Their advantages are not merely additive but synergistic, emerging from the tailored physicochemical environment at the heterointerfaces, which can be engineered to concurrently govern sulfur electrochemistry and sodium deposition kinetics. Here, the multifunctionalities of heterostructured materials for sulfur electrochemistry and Na deposition are detailed (Figure 3).

### 3.1 Regulating Adsorption Sites for Polysulfides and Sodium Ions

The engineered interfaces at a heterostructure material provide a high density of tunable adsorption sites, a principle critical for stabilizing both sulfur cathodes and sodium anodes by controlling the interactions with key species: polysulfides at the cathode and sodium ions at the anode.

At the sulfur cathode, this manifests as robust chemisorption sites for metastable polysulfides. The built‐in electric field arising from Fermi‐level alignment at the interface induces a directional redistribution of interfacial charge to create localized polar surfaces [53, 54, 55]. These polarized regions exert a strong electrostatic pull on the anionic S_x_^2−^ species, enabling rapid initial capture to immobilize these intermediate products. More importantly, the structural defects (e.g., vacancies, lattice distortions, and unsaturated coordination sites, etc.) generated by lattice mismatch of different phase components can serve as highly active centers to form stronger chemical bonds with polysulfides (e.g., Na─S, metal─S, or Lewis acid‐base interactions). This is able to significantly lower the adsorption energy and thus ensure the firm anchoring of polysulfides, thereby effectively suppressing polysulfide dissolution and shuttling.

At the sodium anode, an analogous strategy is employed to create uniform and thermodynamically favorable sodiophilic sites. By incorporating components with intrinsic affinity for sodium ions (e.g., alloying metals or compounds with suitable orbital hybridization), the heterointerfaces are able to exhibit optimized nucleation sites for sodium ions [56, 57, 58]. These sites effectively reduce the nucleation barrier for sodium deposition, guide a uniform Na^+^ flux, and promote dense and lateral plating morphology. The rationally regulated adsorption of sodium ions at the interfaces is fundamental to preventing random dendrite initiation and achieving planar growth.

### 3.2 Enhanced Catalytic Activity for Sulfur Conversion and Sodium Deposition

The catalytic potency of heterointerfaces is pivotal in accelerating the kinetics of the multistep sulfur redox reactions and the sodium deposition/stripping process, both of which are often kinetically sluggish.

For sulfur conversion, the interfacial electronic coupling modifies the local density of states, thus creating catalytically optimal sites [59, 60, 61]. The built‐in electric field facilitates directional electron transfer from the conductive phase to the adsorbed sulfur species to significantly lower the energy barriers for critical bond‐breaking (S─S) and bond‐forming (Na─S) steps. This catalysis is particularly crucial for the solid‐state conversion of Na_2_S_2_ to Na_2_S, a rate‐limiting step in sulfur reduction, ensuring rapid and complete conversion of soluble intermediates and improving sulfur utilization.

For sodium deposition, the catalytic function addresses the high activation barriers of Na^+^ desolvation and charge transfer. Active sites at the heterointerfaces can catalyze the desolvation process and reduce the subsequent electron transfer barrier, consequently realizing a lower nucleation and plating overpotential [62, 63, 64]. The enhanced kinetics can stabilize the operation of sodium anodes at higher current densities and mitigate issues related to concentration polarization, which is a key driver of unstable dendritic growth.

### 3.3 Broadened Selectivity in Reaction Pathway and Interface Engineering

The compositional flexibility of heterostructure materials allows for the selective regulation of complex reaction pathways at the cathode and the targeted engineering of the electrode/electrolyte interface at the anode.

At the sulfur cathode, different interfacial components can be designed to preferentially catalyze specific steps in the multi‐electron sulfur reduction, such as the conversion from long‐chain conversion to short‐chain sulfide nucleation and the reverse oxidation process. This functional cooperation ensures a smoother and more reversible reaction pathway, thus preventing the accumulation of irreversible discharge products (e.g., insoluble Na_2_S_2_) that lead to the formation of dead sulfur and thus the capacity decay [65, 66, 67].

At the sodium anode, the selectivity focuses on concurrently guiding sodium deposition and stabilizing the SEI layer. A heterostructure material can integrate a sodiophilic phase to direct uniform plating with an ion‐conductive phase to modulate the SEI formation [68, 69, 70]. This composite interface favors the in situ formation of a homogeneous, mechanically strong, and ion‐permeable SEI that is rich in beneficial inorganic components and therefore effectively passivates the anode to block dendrite penetration.

### 3.4 Promoting Integrated Charge Transport and Mechanical Stability

Heterostructures intrinsically optimize mass and charge transport while providing essential mechanical reinforcement, a paramount requirement for both high‐sulfur‐loading cathodes and long‐cycle‐life anodes.

At the sulfur cathode side, the integration of a highly conductive phase (e.g., graphene, MXenes, or carbon nanotubes) establishes a percolating network for rapid electron transport throughout the composite [71, 72, 73]. Simultaneously, the interfacial architecture and associated porosity facilitate electrolyte infiltration and Na^+^ diffusion. This dual‐conduction framework is vital for achieving high rate capability and high areal capacity under practical electrode conditions.

At the sodium anode side, the mechanical properties of heterostructure materials are critical. An ideal interfacial layer or host must be chemically stable and mechanically robust to resist large volume changes of sodium metal upon stripping/platting as well as maintain intimate electrical contact. Heterostructures can be designed with a rigid‐yet‐flexible character, where a high‐modulus component (e.g., carbon scaffolds and ceramic particles) suppresses dendrite penetration and provides structural integrity while a softer or more ductile component accommodates strain and maintains interfacial contact. This integrated design enforces a uniform ionic flux and current density, which is the cornerstone of stable and dendrite‐free cycling.

The functional advantages of heterostructure materials generally stem from a universal design principle: programmable interfaces. The same core strategies, involving engineering built‐in electric fields, creating defective active sites, optimizing electronic structures for catalysis, and integrating complementary phases for transport and stability, are adeptly applied to solve the distinct yet related problems at the sulfur cathode and the sodium anode. Such bifunctionality makes heterostructure materials a useful solution that is capable of synchronously reinforcing the most vulnerable components in RT Na‐S batteries

## 4 Design Principles of Heterostructure Materials

**FIGURE 4:**
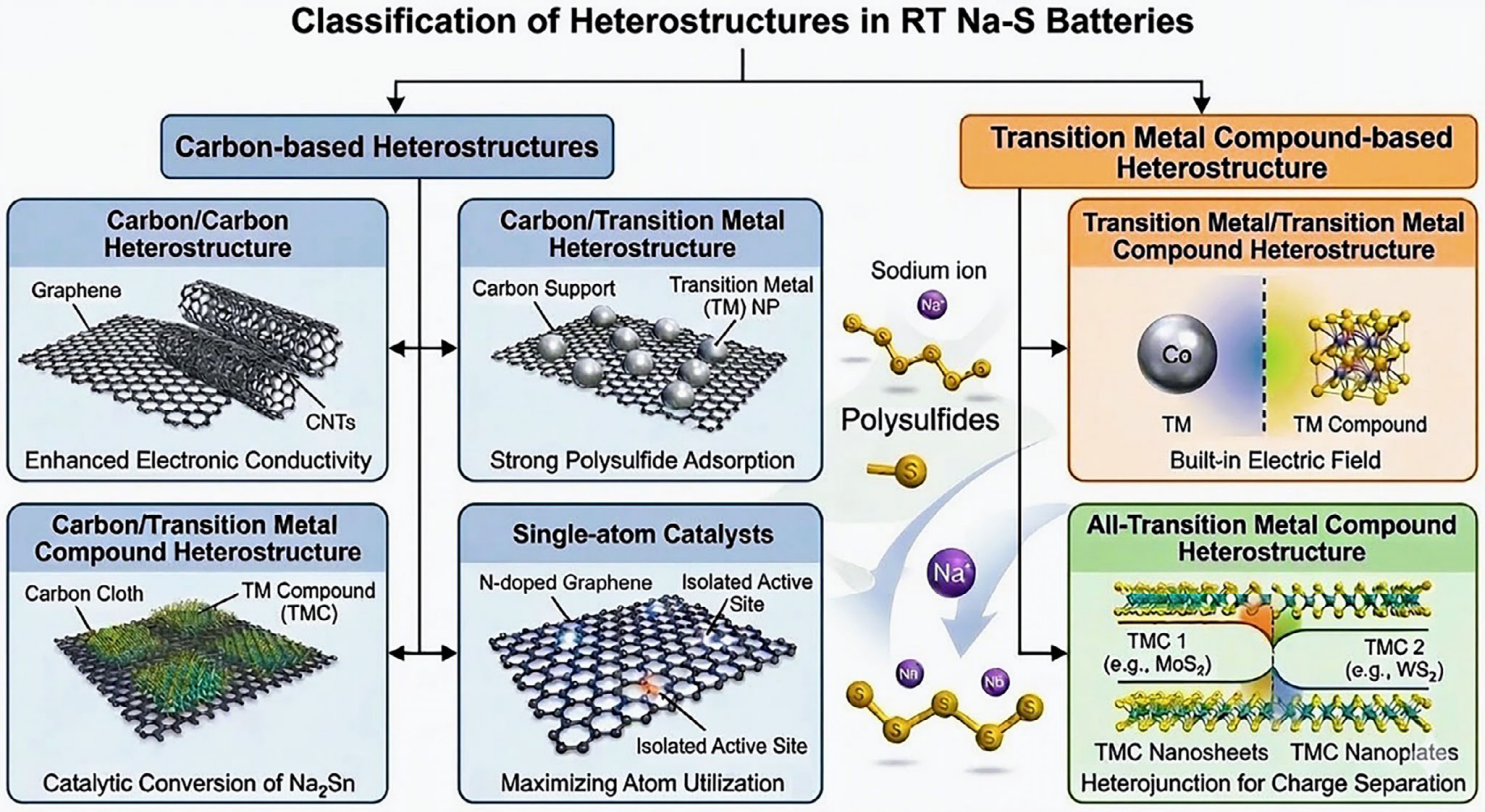
A summary of heterostructure types for RT Na‐S batteries.

The design principle of heterostructure materials for RT Na‐S batteries fundamentally stems from a targeted response to functional requirements of sulfur conversion and sodium deposition. Therefore, heterostructure materials design must simultaneously meet three functional requirements: strong adsorption and confinement, efficient catalytic conversion, and rapid electron/ion transport. The following discussion will focus on the integration of different phase components to realize the coupling of these required functionalities (Figure 4).

### 4.1 Carbon‐Based Heterostructures

A carbon‐based heterostructure refers to a hybrid formed by tightly combining different carbon‐based materials or carbon with other materials at the nanoscale. These heterostructure materials are more than just a simple physical blend and are integrated through chemical bonds or strong interfacial interactions. Common types include carbon/carbon heterostructures, carbon/transition metal heterostructures, and carbon/transition metal compound heterostructures. Single‐atom catalysts (SACs) are also considered a special and important category within this range since the single‐atom sites have strong chemical interactions with the substrate materials such as carbons and MXenes.

#### 4.1.1 Carbon/Carbon Heterostructure

Carbon/carbon heterostructures are integrated systems formed by combining at least two kinds of carbon materials with different structural characteristics, such as dimension, crystal structure, type of heteroatom doping, or pore morphology. The key design principle is to utilize the inherent differences between carbon allotropes or their derivatives. By creating an electron structure gradient and achieving physical structural synergy at their interfaces, these carbon/carbon heterostructures can overcome the limitations of single carbon materials in terms of adsorption activity, charge transport, and structural stability [74, 75, 76].

This design allows them to precisely meet the key requirements in RT Na‐S batteries: (1) effectively trapping polysulfides, enabling fast charge transfer, (2) buffering volume expansion, and (3) maintaining structural stability over long‐term cycling. Because of their fully carbon‐based composition, carbon/carbon heterostructures usually featureby high conductivity, good compatibility, and low cost. These advantages give them unique potentials for applications in RT Na‐S batteries that require high‐rate capability and long cycle life.

#### 4.1.2 Carbon/Transition Metal Heterostructure

In the carbon/transition metal heterostructure system, a carbon material often serves as the functional support, while transition metals act as the active component in the form of clusters or nanoparticles. They are tightly combined through precise interface engineering, where there is tight chemical bonding between the carbon skeleton and transition metal nanoparticles [77, 78, 79]. Typically, the pyrolysis of metal‐contained organic matters (e.g., metal–organic frameworks) under an inert atmosphere can produce transition metal clusters wrapped by localized graphitized carbon layers. Under high temperature, the carbonized organic ligands act as a robust reducing agent, driving the aggregation of metallic elements into highly active nanoparticles or clusters. In a reciprocal dynamic, these in situ formed transition metals serve as catalysts to promote the localized graphitization of the adjacent carbon matrix. This unique confinement architecture not only effectively prevents the severe agglomeration and dissolution of the active metal sites, but also significantly enhances interfacial electron transfer kinetics and structural stability for subsequent electrochemical applications.

This approach directly addresses the four major challenges of the sulfur cathode in RT Na‐S batteries: (1) highly conductive carbon networks and metal can improve the electronic conductivity of sulfur cathodes, (2) the polarized metal clusters can chemically anchor polysulfides to inhibit their shuttling behaviors, (3) fine porous carbon structures can accommodate the large volume changes of active materials during cycling, (4) the tight coupling between two components produce rich heterointerfaces to enrich active sites.

#### 4.1.3 Carbon/Transition Metal Compound Heterostructure

The design of carbon/transition metal compound heterostructures is similar to the aforementioned carbon/transition metal heterostructures; however, there is stronger chemical bonding between carbon materials and transition metal compounds at the nanometer or even atomic scale. These transition metal compounds include oxides [80, 81], sulfides [82, 83, 84], selenides [85], tellurides [86], carbides [87, 88], nitrides [89], and phosphides [90], etc. These compounds can be in situ generated during the pyrolysis or through further treatment of transition metals. The most direct advantage of employing transition metal compounds to design heterostructure materials is that they can offer abundant polar sites to immobilize sulfur species and compensate for the poor chemical affinity of carbons.

More importantly, the transition metal compounds have excellent catalytic ability for sulfur electrochemistry to mediate fast sulfur conversion. This synergy not only improves the trapping and confinement of intermediates but also accelerates their phase transition kinetics during redox reactions. Thus, such heterostructures exemplify a rational material design that combines conductivity, adsorption, and catalysis in a single integrated platform, making them a highly promising configuration for high‐performance sulfur‐based batteries.

#### 4.1.4 Single‐Atom Catalysts

SACs epitomize the ultimate refinement of heterostructure design, where the conventional interface between two phases is refined to the atomic scale, that is, discrete catalytically active metal atoms (e.g., Fe, Co, Ni, Mo) anchored on a conductive carbon substrate constitute well‐defined atomic heterostructures [91, 92, 93]. This architecture leverages the profound metal‐support interaction, typically through coordination with heteroatoms like nitrogen, to induce significant charge redistribution and create a powerful localized electric field. The resulting electronic modulation endows the metal center with an optimal adsorption strength for polysulfides, following the Sabatier principle, thereby transforming each atom into an ultra‐efficient site for chemisorption and catalysis.

For sulfur conversion, this means not only the robust anchoring of polysulfides via metal‐S bonding but also a dramatic reduction in the energy barriers for breaking S─S bonds and forming Na‐S bonds. The atomic‐level sites catalyze the sulfur conversion kinetics decisively, shorten the lifetime of intermediates, and fundamentally curtail the shuttle effect. Concurrently, this atomic interface engineering exerts an equally transformative influence on the sodium metal anode. The uniformly dispersed electron‐tuned metal atoms serve as ideal sodiophilic nucleation sites, significantly lowering the nucleation overpotential and guiding a homogeneous Na^+^ flux [94, 95, 96]. This promotes dense planar sodium deposition and suppresses dendritic growth. Furthermore, the stable covalent anchoring of metal atoms within the carbon matrix ensures the durability of this modified interface during cycling, contributing to a more uniform and robust SEI. Thus, SACs, as atomic heterostructures, masterfully unify the core design principles of interfacial engineering, encompassing precise electronic tuning, maximized active site exposure, and synergistic host‐guest dynamics. They are able to concurrently address the kinetic and morphological challenges at both electrodes, representing a frontier in the development of high‐energy, long‐cycle‐life metal‐sulfur batteries.

Currently, mainstream strategies for optimizing the catalytic activity of single‐atom via precise manipulation of their coordination environments include (1) introducing low‐electronegativity heteroatoms to break coordination symmetry and modulate the d‐band center, (2) utilizing unsaturated coordination to construct asymmetric electron distributions to enhance chemisorption and activating reactants, (3) constructing linearly interconnected atomic chains to act as electron reservoirs and accelerate electron transfer during redox processes, and (4) employing innovative regulation methods, such as voltage modulation, to induce highly dispersed active single‐atom sites from pre‐catalysts.

### 4.2 Transition Metal Compound‐Based Heterostructure

While carbon‐based heterostructure materials provide a foundational conductive framework, transition metal‐centric heterostructures offer intrinsically stronger catalytic activity and interfacial synergy. These heterostructures mainly include transition metal/transition metal compound heterostructures and all‐transition metal compound heterostructures. They are often at the expense of some conductivity but with superior chemical functionality. These systems move beyond the primary role of carbons as an electron highway and instead construct the entire active interface from inherently polar and redox‐active components.

#### 4.2.1 Transition Metal/Transition Metal Compound Heterostructure

Transition metal/transition metal compound heterostructures represent a fundamental and highly effective design, in which a metallic phase (e.g., Co, Ni, Fe) is intimately integrated with a metal compound phase (e.g., sulfide, phosphide, oxide). These compounds may be the corresponding compounds of the metal itself or the compounds of other metals [97, 98]. The main advantage of this architecture lies in the creation of a Mott‐Schottky‐type interface, where the significant difference in Fermi levels between the metal and the semiconductor‐like compound drives spontaneous electron transfer until equilibrium is reached [55]. This process establishes a strong permanent built‐in electric field at the interface. The metallic component serves as an ultra‐conductive electron highway, rapidly delivering electrons to the reactants. Simultaneously, the compound phase with a modulated electronic structure exhibits enhanced polarity and optimized d‐band centers, due to the interfacial charge transfer.

This synergy results in exceptionally strong chemisorption for polysulfides and significantly lowered energy barriers for their conversion. For instance, in a Co/CoS_2_ heterostructure, the metallic Co rapidly transfers electrons to the CoS_2_ surface, where the enriched electron density activates sulfur species and facilitates S─S bond cleavage. This design directly addresses the conductivity limitations of pure metal compounds while amplifying their inherent catalytic properties, offering a more potent and integrated “conductive‐catalytic” unit, compared to carbon‐supported composites, where the carbon host primarily provides conductivity with limited intrinsic catalytic activity.

As a class of cross‐scale heterostructure catalysts, high‐entropy alloys are endowed with abundant and tunable local heterointerfaces by their four core characteristics: high mixing entropy, severe lattice distortion, sluggish diffusion, and the cocktail effect. Furthermore, compared to transition metal alloys and compounds, high‐entropy alloys exhibit superior electrical conductivity, greater multidimensional tunability, and higher stability, establishing them as a highly promising heterostructure catalyst platform with broad application prospects in metal‐sulfur batteries such as Na‐S systems.

#### 4.2.2 All‐Transition Metal Compound Heterostructure

Building upon this principle, all‐transition‐metal compound heterostructures (e.g., CoS_2_/MoS_2_, Ni_3_S_2_/FeS_2_) represent a more sophisticated evolution, where two distinct compound phases are interfaced. This design moves beyond the metal/compound dichotomy to engineer compound semiconductor heterostructures (Type‐I, II, or III) with precise band alignment [99, 100, 101]. The interfacial interaction is governed by differences in work function, electron affinity, and crystal structure, leading to charge redistribution. The formation of an internal field IS often more complex and tunable than those in simple Mott‐Schottky junctions. The key advantage is the synergistic creation of multi‐functional active sites and strain‐induced catalysis. The lattice mismatch at the coherent or semi‐coherent interface generates localized strain and a high density of defects, which serve as superior active sites for adsorbing and activating reactants. More importantly, the aligned band structures can promote the directed separation and migration of charge carriers under electrochemical polarization. For example, in a heterostructure composed of a reduction‐facile compound and an oxidation‐facile compound, the interface can spatially separate the reduction and oxidation half‐reactions, thereby streamlining the entire sulfur redox pathway and minimizing the formation of obstructive intermediates.

All‐transition‐metal compound heterostructures often exhibit superior structural and chemical stability during cycling, as both phases are electrochemically active and can adapt synergistically to volume changes. When contrasted with carbon‐based heterostructures, these inorganic‐inorganic interfaces offer vastly higher density of catalytically active atoms and stronger polar interactions, albeit often requiring integration with a conductive matrix like carbon for optimal rate performance [72, 83, 102]. Thus, they exemplify a design method focused on maximizing interfacial chemical activity and catalytic specificity per unit volume, complementing the carbon‐centric designs that prioritize macro‐scale conductivity and mass transport.

## 5 Practical Applications of Heterostructure Materials

The exceptional interfacial properties and multifunctionality of heterostructure materials transcend fundamental scientific appeal and provide a versatile toolkit for pragmatic engineering solutions across key battery components. Moving from theoretical mechanisms to device‐level implementation, heterostructure materials design principles are being strategically deployed to construct high‐performance hosts materials for sulfur and sodium, engineer functional interface layers for cathode interlayers and anode interface layers, and reinvent current collectors. In each application, the central idea remains consistent, that is, to create tailored interfaces that actively regulate the complex sulfur electrochemistry and sodium deposition behavior.

However, the specific architectural and property requirements differ markedly. As hosts, heterostructure materials must integrate confinement, catalysis, and conduction within a porous scaffold. As interface layers, their priority shifts toward serving as selective ionic/electronic barriers and catalytic filters. As advanced current collectors, they function as monolithic multifunctional substrates that eliminate inactive components. This chapter systematically summarizes these critical applications and elucidates how heterostructure engineering is directly translated into tangible performance enhancements, with a goal of bridging the gap between nanoscale interface science and the realization of practical high‐energy‐density RT Na‐S batteries.

### 5.1 Host Materials

The rational design of sulfur host materials is paramount to harnessing the theoretical capacity of sulfur while mitigating its intrinsic challenges. Heterostructure engineering offers a sophisticated strategy to construct multifunctional hosts where the interface itself becomes the active center for polysulfide management. The design principles evolve across different material combinations, each offering a unique set of advantages.

#### 5.1.1 Sulfur Hosts

**FIGURE 5:**
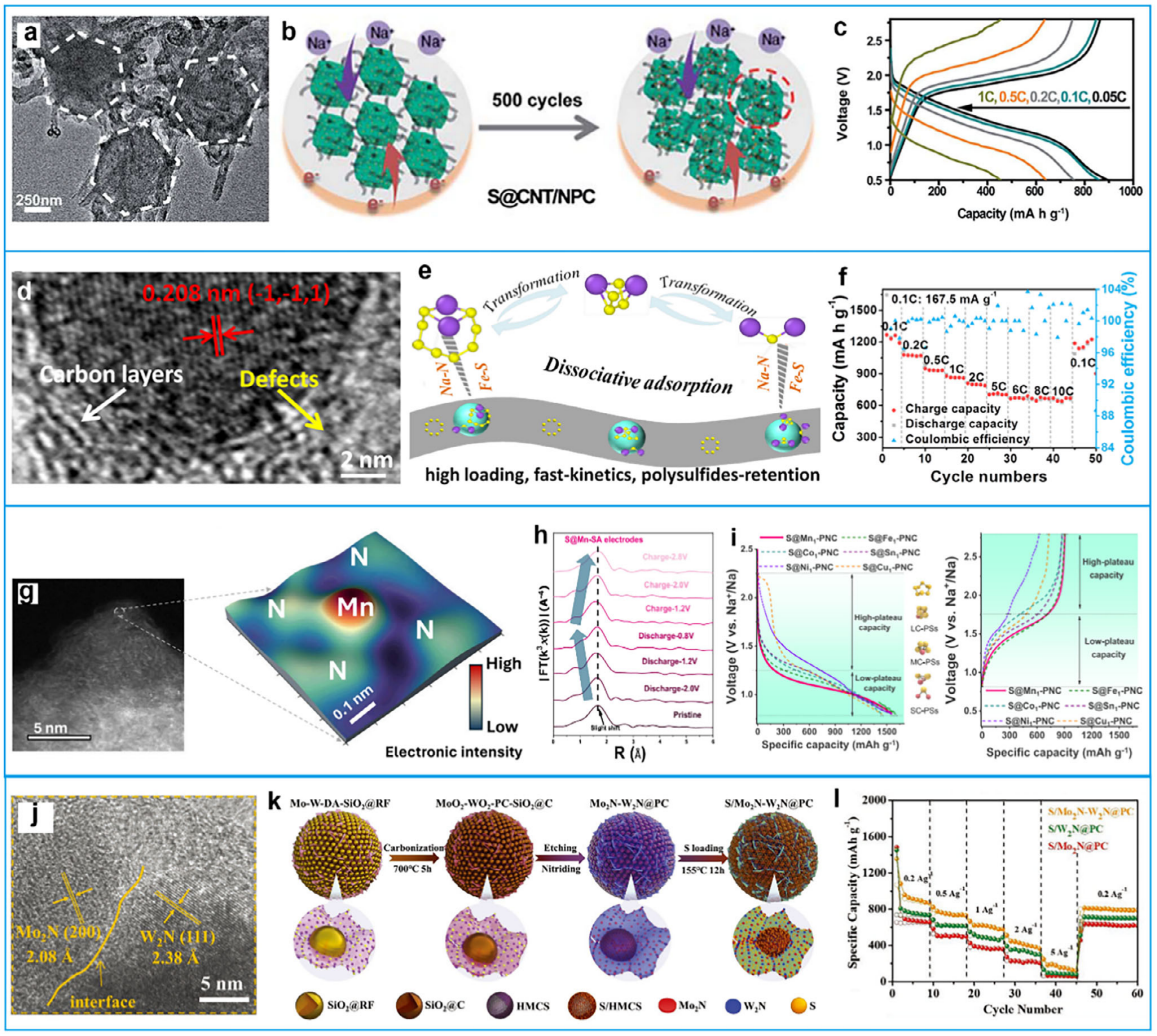
Heterostructured hosts for sulfur cathodes in RT Na‐S batteries. (a) SEM image of the S@CNT/NPC composite. (b) Schematic illustration of the S@CNT/NPC electrodes during cycling. (c) Discharge‐charge profiles of the S@CNT/NPC composite at various rates. Reproduced with permission [103, 104]. Copyright 2019, Royal Society of Chemistry. (d) HRTEM image of Fe_3_N‐NMCN composite. (e) Function of the S@Fe3N‐NMCN cathode. (f) Rate the performance of the Fe_3_N‐NMCN composite. Reproduced with permission [19]. Copyright 2021, Springer‐Nature. (g) High‐resolution HAADF image of Mn_1_@PNC, together with the three‐dimensional (3D) profile of the local structure of the Mn_1_ atoms. (h) *k* ^3^‐weighted FT‐EXAFS curves of S@Mn_1_‐PNC in R‐space during the initial discharge and charge processes. (i) Initial discharge and charge profiles. Reproduced with permission [120]. Copyright 2024, Springer‐Nature. (j) HRTEM images of the spherical Mo2N–W2N@PC superstructure. (k) Schematic illustration of the preparation of the spherical S/Mo_2_N–W_2_N@PC superstructure. (l) Rate the performance of the spherical S/Mo_2_N–W_2_N@PC superstructure. Reproduced with permission [133]. Copyright 2021, Wiley‐VCH.

The applications of heterostructured sulfur hosts in RT Na‐S batteries have evolved from the carbon‐carbon heterostructures at the early stage. This type of heterostructured hosts involves the integration of different allotropic or morphological carbon forms, such as graphene/carbon nanotube hybrids, N‐doped porous carbon/graphene composites, or covalent organic framework‐derived hetero‐carbons, etc [103, 104]. The primary advantage lies in creating synergistic conductive networks with hierarchical porosity. For instance, Xu et al., designed a 3D railway‐like carbon host for Na‐S batteries, where porous carbon polyhedrons (NPCs) were concatenated by CNTs, as displayed in Figure 5a. The porous carbon polyhedrons could provide accommodation space to active materials, while the CNTs could serve as bridges to transport electrons. Such a design could also help to maintain the cathode integrity during the cycling process (Figure 5b). Therefore, the 3D sulfur cathode demonstrated a very low capacity decay rate of 0.064% per cycle after 500 cycles at 0.5C and obtained great rate performance (Figure 5c). Moreover, the heteroatom doping (N, B, S, P) at the junction of different carbon phases creates local charge polarization, transforming an otherwise non‐polar carbon surface into a medium‐polarity interface capable of moderately binding polysulfides via dipole‐dipole and Lewis acid‐base interactions [105, 106, 107]. For example, a phosphor‐doped carbon network (phos‐C) was proposed by Wang et al., to serve as an efficient sulfur host for RT Na‐S batteries [105]. The phosphor atoms were proved to increase polar sites, intensify the adsorption for polysulfides (Na_2_S_n_), and promote their catalytic conversion. As a result, the P‐doped sulfur cathode realized a capacity retention of 480 mA h g^−1^ after 2000 cycles at 1C and displayed a superior rate capability of 339 mA h g^−1^ at an ultrahigh rate of 10C. These hosts are excellent in buffering volume expansion, ensuring excellent electrical connectivity, and providing physical confinement, making them foundational scaffolds. However, their polysulfide adsorption strength is often insufficient to completely suppress shuttle under lean electrolyte conditions, necessitating integration with stronger chemisorbing components.

It is the most extensively studied category, where conductive carbon matrices are integrated with polar transition metal compounds encompassing oxides (rGO/VO_2_ [108], CoMoO_4_@rGO [109], S/Nb_2_O_5_‐CNR [22]), sulfides (CoS_2_, MoS_2_) [110, 111], selenides (Fe_3_Se_4_ [112], CoSe_2_ [113]), tellurides (MoTe_2_ [114, 115]), nitrides (Fe_3_N [19], Co_4_N [116], MoN [7]), and carbides (VC [117], MoC [118], Mo_2_C [119]) etc. The carbon guarantees rapid electron transfer and structural resilience, while the metal compounds provide abundant chemisorption sites via polar‐polar and Lewis acid‐base interactions. The key advancement is the formation of an intimate heterointerface that often exhibits enhanced catalytic activity beyond either component alone. The charge transfer from carbon to the metal compound (or vice versa) modulates the electronic state of the metal sites, optimizes their d‐band center for polysulfide adsorption, and lowers the activation energy for their conversion. This creates a typical confinement‐conversion synergy, significantly improving sulfur utilization and cycling life, where the carbon host immobilizes sulfur and confines reaction space while the metal compounds chemically anchor and catalyzes the intermediates,. For instance, Qi et al., elaborated nitrogen‐doped multilayer carbons decorated with Fe_3_N quantum dots(Fe_3_N‐NMCN) for Na‐S batteries [19]. The Fe_3_N quantum dots uniformly embedded within the carbon nanofibers were able to offer abundant active sites for sulfur redox reactions (Figure 5d). The strong chemical affinity between Fe_3_N and polysulfides, manifested as the formation of Na─N and Fe─S bonds (Figure 5e), was demonstrated to immobilize polysulfides and accelerate their dissociation, thereby suppressing the shuttle effect. Consequently, the Fe_3_N‐NMCN@S cathode could achieve nearly 100% capacity retention after 2800 cycles at 5C and even deliver excellent rate capability of 658.4 mA h g^−1^ at an ultrahigh current density of 10C (Figure 5f).

Representing the ultimate limit of interface engineering, SACs anchor isolated transition metal atoms (Fe, Co, Ni, Mo) on N‐doped or defective carbon scaffolds via M─N_x_ or M─C coordination. This architecture transforms the host into an array of atomic‐scale and homogeneous reactive centers. Each metal atom, with its unsaturated coordination and quantum‐confined electronic structure, acts as an ultra‐efficient dual‐functional site. It exhibits ideal adsorption strength for polysulfides, following the Sabatier principle, and serves as a supreme catalyst for accelerating both the reduction of long‐chain Na_2_S_n_ and the nucleation of Na_2_S. The uniform distribution of these atomic sites ensures that every polysulfide molecule encounters an identical active interface, maximizing catalytic efficiency and promoting spatially homogeneous sulfur deposition. This leads to exceptionally high active material utilization even at high sulfur loadings and low electrolyte/sulfur ratios. For example, Lei et al., constructed a Mn SAs–modified nitrogen‐doped porous carbon nanosphere (S@Mn_1_‐PNC) for Na‐S batteries, as shown in Figure 5g [120]. The Mn‐N_4_ sites were discovered to enable moderate adsorption strength and rapid electron transfer, as evidenced by Figure 5h. This promoted the direct cleavage of S_8_ molecules into short‐chain polysulfides and thus inhibited the generation of metastable long‐chain polysulfides (Figure 5i). The Mn SA catalyst also possessed high selectivity toward the conversion of short‐chain polysulfide. As such, the elaborate S@Mn_1_‐PNC cathode maintained a relatively high capacity of 344.1 mA h g^−1^ after 3000 cycles at 2 A g^−1^ as well as showed superior rate capability of 576 mA h g^−1^ at 5 A g^−1^. Currently, many kinds of SACs‐based hosts have been reported for RT Na‐S batteries, including Mn [121], V [122], Y [94], Fe [123, 124, 125], Zn [126, 127, 128], Cu [129], Co [130], Pt [131].

Moving beyond carbon, all‐transition metal compound heterostructure materials involve the direct coupling of two different transition metal compounds, such as CoS_2_/MoS_2_, SnS_2_/FeS_2_, or MXene/MoS_2_ hybrids, etc. These inorganic‐inorganic interfaces are designed to maximize intrinsic catalytic synergy and interfacial charge transfer. The built‐in electric field at heterointerfaces is often more intense than in carbon‐based systems. This field drives directional electron flow, dramatically enhancing the kinetics of multi‐electron transfer in sulfur redox reactions. Furthermore, the lattice mismatch at the coherent interface generates strain and defects that are themselves highly catalytic. These hosts typically offer the strongest polysulfide chemisorption and the highest density of catalytically active sites per unit volume. A typical example is that Reddy et al., in situ grew TiO_2_ nanoparticles onto Ti_3_C_2_ MXene layers to fabricate a TiO_2_‐Ti_3_C_2_ heterostructure host for Na‐S batteries [132]. The TiO_2_ nanoparticles were uniformly distributed between the Ti_3_C_2_ layers to expose abundant polar sites, and meanwhile, high catalytic activity could be endowed by Ti_3_C_2_ nanosheets. This heterostructure design realized smooth functional integration to ensure effective immobilization of polysulfides, rapid Na^+^ diffusion, and catalytic sulfur conversions. As a result, the TiO_2_‐Ti_3_C_2_@S cathode could retain a reversible capacity of 255.2 mA h g^−1^ after 1500 cycles at 1C. Their challenge lies in intrinsic low electronic conductivity, which is often mitigated by designing them as nanoarchitectures (e.g., ultrathin nanosheets, hierarchical flowers) or by hybridizing them with a minor amount of conductive carbon. For example, Zhang et al., embedded Mo_2_N‐W_2_N heterostructures (Mo_2_N‐W_2_N@PC) into superstructured porous carbon spheres for Na‐S batteries [133]. This heterostructure combined the respective advantages of Mo_2_N and W_2_N to enhance the electronic conductivity of the heterostructure catalyst. Besides, its abundant heterointerfaces were able to facilitate electron/ion diffusion as well as reinforce catalytic activity for polysulfide conversions. Finally, the S/Mo_2_N‐W_2_N@PC cathode retained a reversible capacity of 517 mA h g^−1^ after 400 cycles at 1 A g^−1^ and exhibited excellent rate capability of 417 mA h g^−1^ at 2 A g^−1^. So far, many transition metal compounds have been considered to construct heterostructured materials for Na‐S batteries, including oxides (e.g., TiO_2_ [134] and CeO_2_ [135]), chalcogenides (e.g., FeS_2_ [137], CoS_2_, and CoSe_2_ [139]), carbides (MoC, W_2_C [136], MXene [138]), nitrides (e.g., TiN), and so on.

#### 5.1.2 Sodium Hosts

The applications of heterostructures in sodium hosts need to consider the regulation of sodium deposition at the source. Unlike sulfur hosts that primarily manage intermediates, sodium hosts must contend with the dynamic, solid‐state processes of nucleation, plating, stripping, and the immense associated volume changes. Heterostructure materials, through their tunable interfacial properties, offer innovative solutions by creating spatially and chemically defined environments that guide sodium behavior. The design concept progresses from frameworks offering structural stability to those providing atomic‐level electrochemical regulation.

**FIGURE 6:**
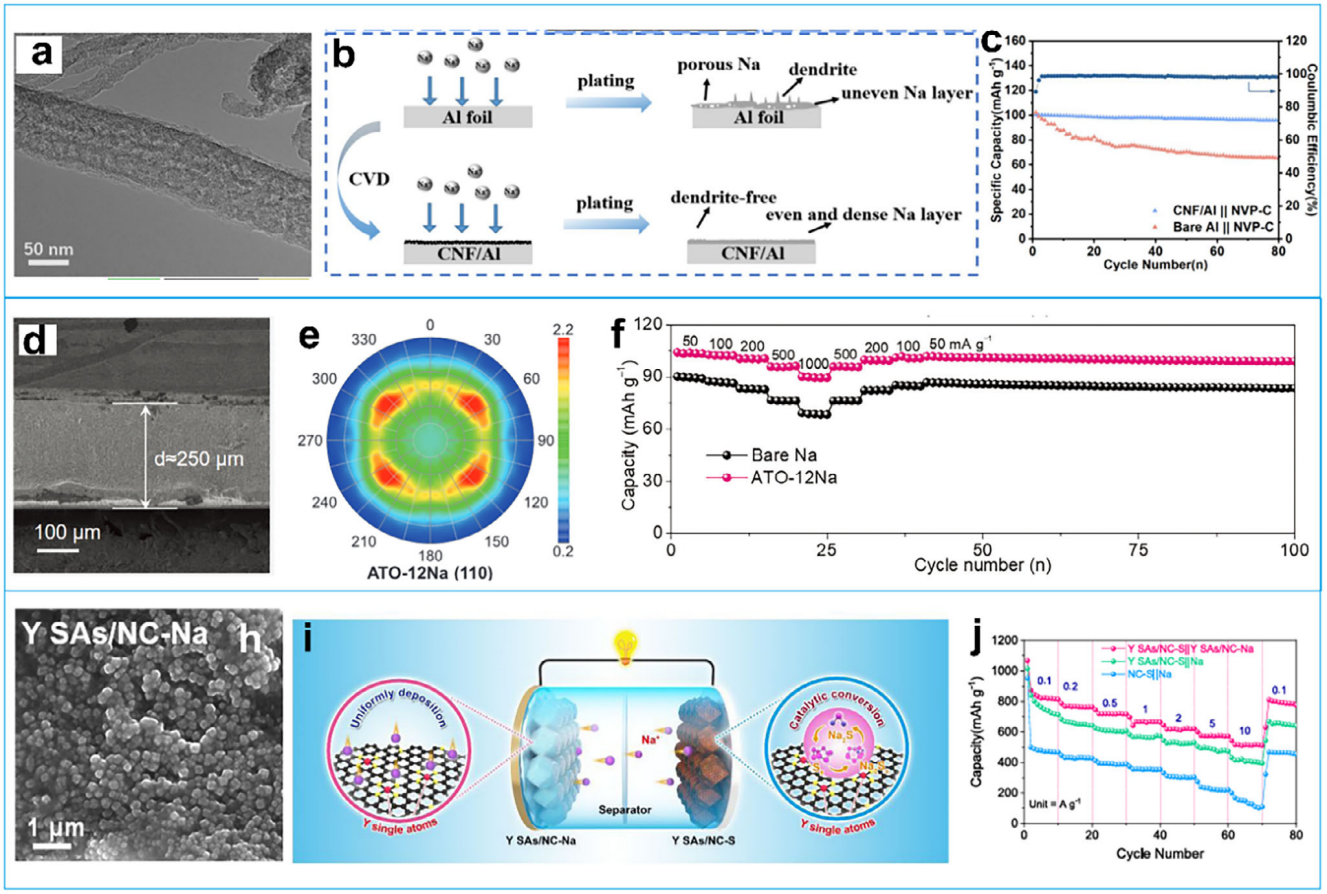
Heterostructure hosts for sodium anodes in RT Na‐S batteries. (a) HRTEM image of CNFs. (b) Na plating behavior on Al foil and CNF/Al electrodes. (c) The cycling performance of the CNF/Al composite. Reproduced with permission [140]. Copyright 2025, Springer US. (d) SEM image of the ATO‐12Na. (e) (110) plane pole figures of the ATO‐12Na. (f) Rate the performance of the ATO‐12Na. Reproduced with permission [145]. Copyright 2024, Wiley‐VCH. (h) SEM image of the Y SAs/NC‐Na anodes after cycling. (i) Schematic illustration of the Y SAs/NC‐S||Y SAs/NC‐Na full cell. (j) Rate performances of the Y SAs/NC‐S||Na composite, respectively. Reproduced with permission [94]. Copyright 2022, American Chemistry Society.

In terms of real applications, three‐dimensional carbon heterostructures, such as interwoven carbon nanotube/graphene foams, N‐doped carbon fiber networks, or hierarchically porous carbon spheres, primarily serve as mechanically robust and sodiophilic scaffolds [63, 140, 141]. Their high surface areas and conductive interconnected networks lower the local current density, which is fundamental to delaying dendrite initiation. The practical advancement lies in their ability to confine sodium deposition within their porous cavities, thereby accommodating volume expansion in the vertical direction and preventing electrode‐level deformation. A case study is that Luo et al., constructed a porous carbon nanofiber scaffold on the surface of Al foil (CNF/Al) via chemical vapor deposition to function as a current collector for sodium metal anodes, as presented in Figure 6a [140]. This 3D porous structure provided abundant Na^+^ transport pathways, dispersed the local current density, regulated the deposition behavior of sodium metal, and thereby effectively inhibited dendrite growth (Figure 6b). Electrochemical performance evaluation showed that the CNF/Al electrode operated for 300 cycles with an average Coulombic efficiency of 99.7% at 1 mA cm^−2^ and 1 mA h cm^−2^. Furthermore, the CNF/Al || NVP‐C full cell was able to keep a capacity retention of 95.7% after 80 cycles at 1C (Figure 6c). This simple and scalable electrode preparation method offers promising prospects for the large‐scale application of sodium metal anodes. When heteroatoms (N, O, S) or functional groups are selectively introduced into carbons, they create localized sodiophilic sites that guide uniform sodium nucleation. The key application metric for these hosts is their success in achieving stable cycling at moderate current densities (0.5–2 mA cm^−2^) with high areal capacity (3–5 mA h cm^−2^), often demonstrated in symmetric cell configurations. However, their limited intrinsic affinity for sodium and the risk of uncontrolled pore filling remain challenges under deep cycling conditions.

The carbon‐transition metal compound hybrid hosts can address the limitations of pure carbon by incorporating polar, often alloying transition‐metal compounds such as Sn/Sb/Bi‐based sulfides, oxides, or phosphides onto carbon scaffolds. In practice, materials like SnS_2_ nanosheets anchored on carbon cloth or Sb nanoparticles embedded in carbon foam function as composite hosts with graded functionality [142, 143, 144]. The transition metal compound component, through alloying reactions or strong adsorption, provides thermodynamically favorable and homogeneous nucleation sites, significantly reducing the nucleation overpotential. The carbon matrix then ensures continuous electron transport and buffers the strain from the alloying/dealloying and plating/stripping processes. Recently, Li et al., uniformly embedded antimony‐doped tin oxide (ATO) nanoparticles into the sodium metal matrix to obtain a composite anode with Na(100) texture (ATO‐12Na), as displayed in Figure 6d [145]. They found that the Na(100) texture not only facilitated the formation of an anion‐derived inorganic‐rich SEI layer on the surface of the ATO‐12Na composite anode but also effectively induced uniform and horizontal sodium deposition during the pre‐deposition stage (Figure 6e). Benefiting from the intrinsic sodiophilicity of the Na(100) texture and the high sodiophilicity of ATO active sites, this composite anode possessed greatly enhanced interfacial compatibility and excellent sodium plating/stripping stability. Consequently, the ATO‐12Na symmetric cell stably cycled for over 1400 h at a current density of 2 mA cm^−2^. Furthermore, the full cell assembled with a Na_3_V_2_(PO_4_)_3_ cathode maintained a high capacity retention of 80.7% after 4500 cycles at 500 mA g^−1^ and delivered superior rate performance (Figure 6f). The effectiveness of these hosts is their demonstrated ability to maintain low‐voltage hysteresis and high Coulombic efficiency (>99.5%) over extended cycles in full cells, as the strong interfacial bonding between sodium and the transition metal compound prevents detachment and “dead sodium” formation. Their design is particularly effective for pre‐sodiation or in composite anodes for anode‐free configurations.

The applications of SACs to sodium hosts is an emerging frontier focused on electrochemical catalysis at the nucleation stage. When atomic metals (e.g., Zn, Cu, Co) anchored on N‐doped carbon are coated on separators or integrated into hosts, they act as a catalytic interface that modifies the Na^+^ double layer and reduces the activation barrier for charge transfer [96, 146, 147]. In practical operation, this translates to an exceptionally low and stable nucleation overpotential, even at high current densities. For example, Zhang et al., designed a yttrium SA‐doped nitrogen‐doped carbon dodecahedron (Y SAs/NC) to simultaneously regulate sulfur conversion and sodium deposition in RT Na‐S batteries [94]. Figure 6g shows the microscopic image of the as‐generated sodium anode. The Y‐N_4_ sites exhibited excellent intrinsic sodiophilicity to induce uniform sodium deposition and thus inhibit dendrite growth (Figure 6h). Ultimately, the Na‐S full cell assembled with the Y SAs/NC‐S cathode and Y SAs/NC‐Na anode displayed a high reversible capacity of 822 mA h g^−1^ at 0.1 A g^−1^, maintained a high capacity retention of 97.5% after 1000 cycles at 5 A g^−1^, and demonstrated excellent rate capability of 516 mA h g^−1^ even at 10 A g^−1^ (Figure 6i). The atomic sites can guide a dense mosaic‐like deposition morphology rather than filamentary growth. The most promising application of SACs lies in their ultra‐low mass loading and high activity, enabling the use of ultra‐thin functional coatings on commercial separators to dramatically enhance the cycling stability of standard sodium foil.

### 5.2 Interface Layers

#### 5.2.1 Interlayers Toward Sulfur Cathodes

Interlayers toward sulfur cathodes represent a strategy involving less invasive intervention, compared to direct sulfur host engineering. By positioning a heterostructured interlayer between the cathode and separator (or directly coating it onto the separator), a secondary adosprtive/catalytic shield is introduced to the cell. This shield serves the dual purpose of intercepting and diffusing polysulfides and facilitating their conversion. Their design principles and practical applications of these interfacial layers evolve across several heterostructure categories, each offering distinct advantages and performance metrics.

**FIGURE 7:**
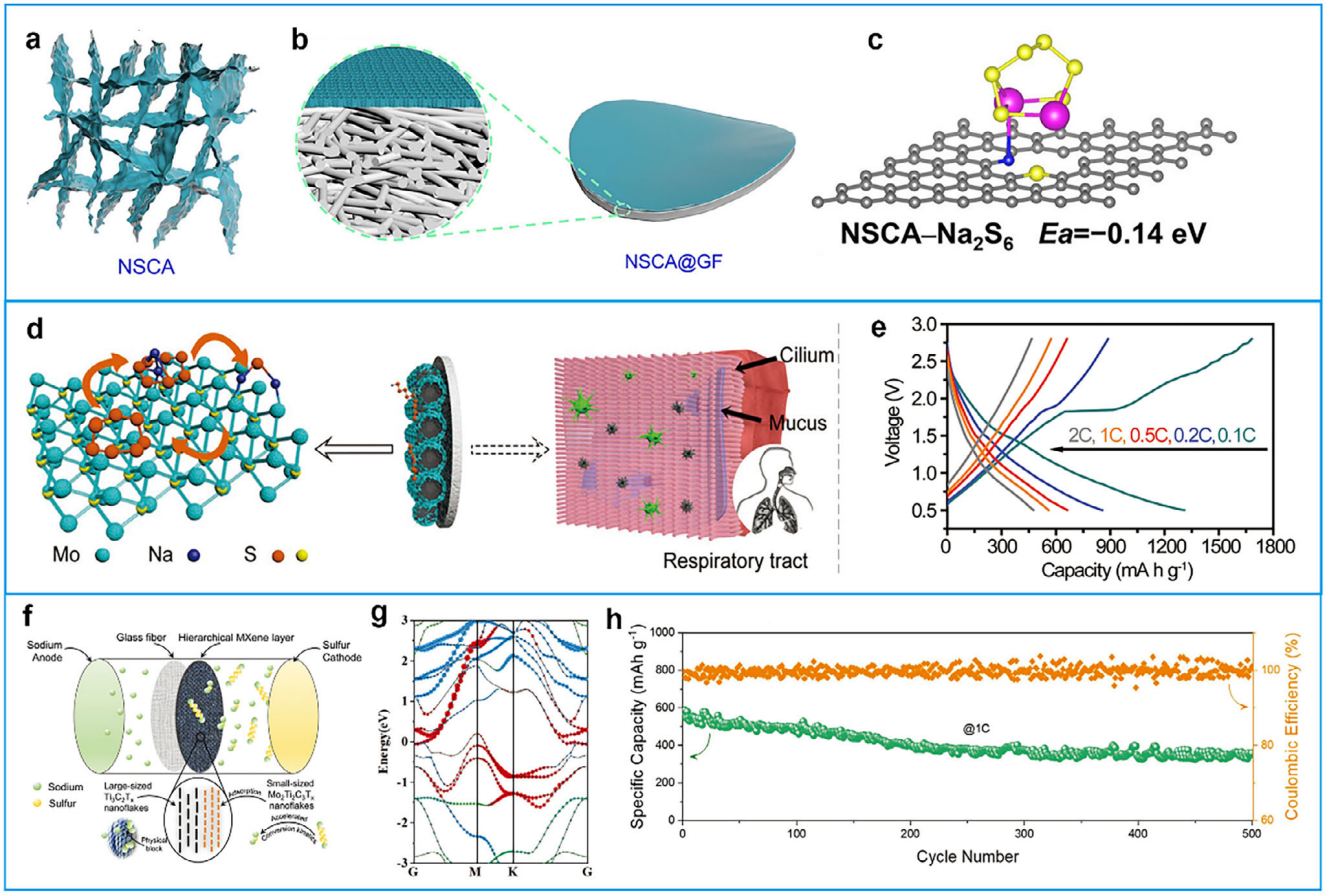
Heterostructure interlayers toward sulfur cathodes for RT Na‐S batteries. Schematic illustration of the morphological characterization of (a) NSCA and (b) NSCA@GF. (c) The optimized adsorption configurations and charge density differences of the NSCA substrates. Reproduced with permission [148]. Copyright 2023, Wiley‐VCH. (d) Schematic diagram of the absorption and respiratory cilia. (e) Voltage profiles of the S@HCS/MoS_2_ composite. Reproduced with permission [155]. Copyright 2019, Wiley‐VCH. (f) Schematic of the configuration of the RT Na‐S battery system with the hierarchical MXene interlayer on the glass fiber separator. (g) Band structure and density of states (DOS) of Mo_2_Ti_2_C_3_T_x_. (h) The cycling performance of the GF@Hybrid MXene separator composite. Reproduced with permission [152]. Copyright 2023, Wiley‐VCH.

The applications of carbon–carbon heterostructures as interlayers primarily focus on establishing a highly conductive and selective barrier for sulfur species. These materials exert their hierarchical porosity to physically block large polysulfide anions while allowing unhindered Na^+^ transport. The primary advantage is that their mechanical flexibility and electrochemical stability enable the fabrication of freestanding interlayer films or ultrathin coatings that add minimal weight or volume to the cell. For example, Yang et al., utilized a self‐assembly method to prepare an N, S‐co‐doped carbon aerogel (NSCA) composed of cellulose nanofibers and graphene oxide (Figure 7a,b) [148]. It was further coated onto a glass fiber separator (NSCA@GF) as a multifunctional interlayer for Na‐S batteries. Its porous network structure with lightweight characteristics and a high specific surface area was helpful to electrolyte infiltration and ion transport. Moreover, the NSCA layer not only acted as a physical barrier to effectively block polysulfide shuttling but also chemically anchored polysulfides and accelerated their reaction kinetics, as suggested by Figure 7c. As a result, Na‐S batteries assembled with the NSCA@GF separator delivered a high reversible capacity of 788.8 mA h g^−1^ after 100 cycles at 0.1C and achieved an ultralow capacity decay rate of only 0.059% per cycle over 1000 cycles at 1C. Furthermore, the introduction of heteroatoms (N, O, S) can also create localized polarized surfaces, enhancing the chemical affinity for polysulfides beyond mere physical confinement [149, 150]. While their polysulfide anchoring strength is moderate, these interlayers are highly effective in improving the sulfur utilization and cycling stability of conventional high‐sulfur‐loading cathodes by providing an auxiliary conductive network and a diffusion barrier.

In contrast, interlayers with carbon‐transition metal compound heterostructures can provide more chemisorption and catalytic sites, which come from surface active sites of transition metal compounds [151, 152, 153, 154]. For instance, Yang et al., constructed a cathode‐separator dual‐barrier system, that is MoS_2_ nanosheet‐decorated hollow carbon spheres (HCS/MoS_2_) were used as the sulfur host (S@HCS/MoS_2_) and building blocks to separator coating layers [155]. The HCS exhibited a uniform hollow spherical morphology for sulfur loading and buffered volume expansion. The MoS_2_ nanosheets were tightly attached to the HCS surface to establish an outer shell approximately 30 nm thick, which not only enhanced the conductivity but also effectively captured polysulfide intermediates through polar adsorption. Figure 7d vividly presents the working mechanism of the interlayer for mediating sulfur species. More importantly, the HCS/MoS_2_‐coated separator further prevented polysulfide from shuttling to the sodium anode. Accordingly, the Na‐S battery assembled with the S@HCS/MoS_2_ cathode and the HCS/MoS_2_‐modified separator delivered a high initial capacity of 1309 mA h g^−1^ at 0.1C, maintained stable capacity over 1000 cycles at 1C, and exhibited excellent rate capability with a capacity of 476 mA h g^−1^ retained at 2C (Figure 7e). The practical appeal of such separator coating layers lies in their effective performance enhancement using relatively simple slurry‐coating processes applicable to commercial separator modification.

Moving beyond carbon, the direct coupling of two transition metal compounds creates interlayers with intense interfacial electric fields and a high surface density of catalytic sites. These inorganic heterostructure materials are engineered for superior electrocatalytic activity and mechanical robustness. A notable example is a hierarchical hybrid MXene interlayer for RT Na‐S batteries, comprising an inner layer of large Ti_3_C_2_T_x_ nanosheets for physical blocking and an outer layer of small Mo_2_Ti_2_C_3_T_x_ nanoflakes for catalytic conversion (Figure 7f–h) [152]. This design enables stable cycling over 200 cycles with 71% capacity retention. Their challenge lies in managing their intrinsic conductivity, which is often addressed by designing them as ultrathin layers or incorporating them into conductive skeletons (e.g., carbons).

#### 5.2.2 Anode Interface Layers

The greatest challenge for sodium metal anodes often resides at its dynamic and unstable interface with the electrolyte. The uncontrolled SEI leads to more severe dendrite growth, dead sodium accumulation, and continuous electrolyte consumption. Constructing an artificial heterostructure‐based interfacial layer directly on the sodium surface offers a proactive strategy to dictate the rules of sodium deposition from the outset. These engineered layers function not merely as passive barriers but as active multifunctional regulators of Na^+^ flux, nucleation behavior, and interfacial chemistry. Such heterostructured interface layers can be evolved through in situ electrochemical formation, pre‐designed chemical composition, and ex situ artificial fabrication, etc.

**FIGURE 8:**
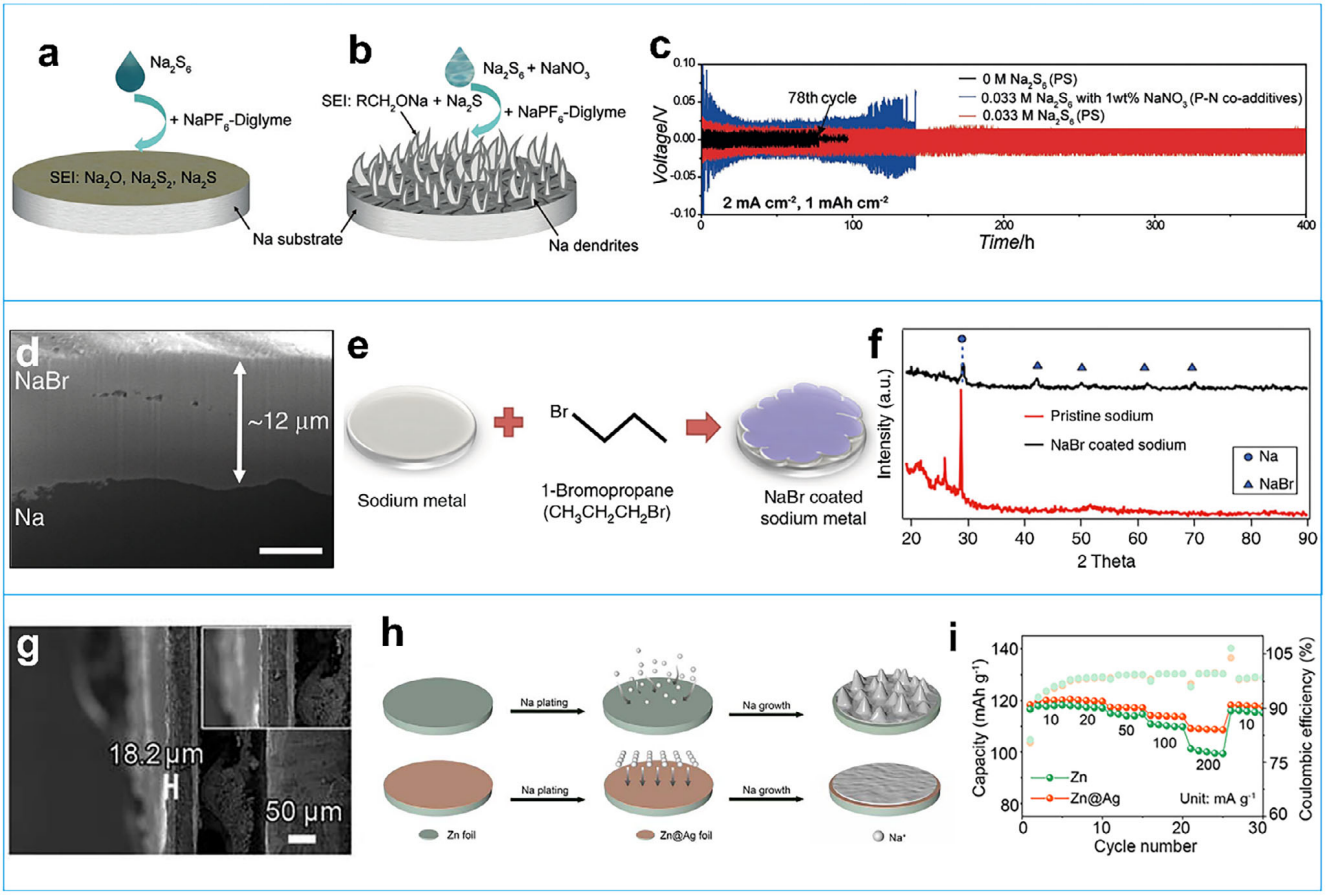
Heterostructure design for anode interface layers in RT Na‐S batteries. The Na surface morphology difference with (a) PS alone and (b) P‐N co‐additives. (c) Galvanostatic cycling of symmetric cells at 5 and 1 mAh cm^−2^. Reproduced with permission [159]. Copyright 2018, Wiley‐VCH. (d) SEM images of the NaBr coat. (e) Schematic showing the procedure used to coat Na with NaBr. (f) XRD analysis of pristine and NaBr‐coated sodium. Reproduced with permission [166]. Copyright 2017, Springer‐Nature. (g) SEM images of the Zn@Ag substrate. (h) Schematic diagram of Na plating on Zn and Zn@Ag substrate. (i) Rate performance of anode‐less battery. Reproduced with permission [170]. Copyright 2019, Royal Society of Chemistry.

The approach of electrochemically inducing heterostructured interphases involves the introduction of specific chemical precursors (additives, co‐solvents, or sacrificial salts) into the electrolyte. They can decompose preferentially on the sodium surface during initial cycles, forming an SEI with inherent heterostructure characters. The decomposition products of different precursors can naturally undergo phase separation, creating nanoscale domains of distinct composition and function within the SEI. For instance, inorganic compounds like NaF, Na_2_O, or Na_3_N provide mechanical strength and high interfacial energy to block dendrites [156, 157, 158], which usually come from additives such as fluoroethylene carbonate, vinylene carbonate, and NaN_3,_ etc. Simultaneously, organic components (e.g., sodium alkoxides, polycarbonates) offer flexibility to accommodate volume changes of sodium metal. The key advantage of this in situ electrochemically induced heterostructure is its conformal and self‐healing nature, since it forms directly and intimately on the evolving sodium surface, even within surface crevices. For instance, Wang et al., demonstrated the crucial role of electrolyte additives in stabilizing the sodium metal anode [159]. By introducing Na2S6 alone into the diglyme electrolyte, a robust SEI was formed that effectively suppressed Na dendrite growth, as illustrated in (Figure 8a). Conversely, the introduction of both Na_2_S_6_ and NaNO_3_ as co‐additives resulted in an unstable SEI, causing severe dendritic and mossy Na growth (Figure 8b). Benefiting from the stable SEI formed by Na2S6 alone, the symmetric cell maintained a highly stable voltage profile for over 400 cycles, showcasing vastly superior cycling stability compared to the cells with co‐additives or no additives (Figure 8c). In fact, the efficacy hinges on the precise selection and ratio of precursors to engineer an SEI with an optimal balance of modulus and toughness. It has been shown that a heterostructured SEI (HSEI) rich in a gradient of NaF nanocrystals embedded in an organic matrix can guide uniform Na^+^ flux, leading to dense sodium plating [160, 161, 162]. The challenge lies in controlling the complex and coupled decomposition reactions to ensure reproducibility and long‐term stability of the formed interfaces.

The method of chemically derived heterostructure interface layers employs direct chemical reactions between a pre‐coated layer and the metallic sodium or the electrolyte to create a stable heterostructured artificial layer. A thin film of a reactive material (e.g., SnCl_4_, Sb_2_O_3_, polyphosphoric acid) is first applied to the sodium foil. Upon contact, a spontaneous chemical reaction occurs, transforming these reactants into a composite layer [163, 164, 165]. A classic example is the chemical reaction of SnCl_4_ with sodium metal, that is, SnCl_4_ + 8Na → 4NaCl + SnNa_4_. It can instantly generate a sodiophilic SnNa_4_ alloy phase embedded within a NaCl‐rich matrix. This creates a built‐in heterostructure, that is, the sodiophilic alloy domains (SnNa_4_, SbNa_3_). It is able to provide lower barrier and homogeneous nucleation sites, while the ion‐conductive yet mechanically robust matrix (NaCl, Na_2_O) ensures fast Na^+^ transport and suppresses dendrite penetration. A case study is that Choudhury et al., demonstrated a simple chemical strategy to create a protective NaBr solid‐electrolyte interphase on sodium metal anodes [166]. By directly reacting the sodium metal with 1‐bromopropane via the Wurtz reaction, a uniform layer of NaBr was synthesized on the anode surface (Figure 8d–f). This method offers precise control over initial interface composition and immediate functionality from the first cycle. The success of this strategy depends on the thermodynamics and kinetics of the reduction reactions. The chosen precursor must react completely and uniformly to form a cohesive adherent layer. The volume change during the conversion reactions must also be managed to avoid the fracture of in situ generated coating layers. Such chemically derived layers are particularly effective in significantly increasing the cycle life of symmetric cells and enabling stable cycling in full cells under moderate current densities and capacities.

In comparison, the most direct approach involves the ex situ fabrication and transfer of a freestanding multicomponent film onto the sodium anode, serving as a ready‐made artificial HSEI. This HSEI is a rationally designed laminate or composite film, often integrating materials with complementary properties. A common design pairs a rigid inorganic top layer (e.g., Al_2_O_3_ and graphene oxide) for mechanical blockade with a soft ion‐conductive bottom layer (e.g., polymer electrolyte and succinonitrile) for ensuring intimate contact [167, 168, 169]. More advanced designs incorporate nanocrystals of sodiophilic metals (e.g., Au and Sn) or ion‐conductive ceramics (Na‐β''‐Al_2_O_3_) within a polymer matrix (e.g., PVDF‐HFP and PAN) to create a true 3D heterostructure [170]. For example, Chen et al., designed a conductive and sodiophilic modified substrate, where an Ag coating layer was sputtered onto a commercial Zn foil to overcome the uneven [170]. The Ag coating could provide enhanced sodiophilicity to lower the nucleation barrier, while its superior electronic conductivity could serve to homogenize the Na ion and electric field distribution. Such a design could also help to maintain the anode's structural integrity, yielding a highly compact and dendrite‐free Na morphology during the deposition process (Figure 8g,h). Therefore, the anode‐less battery coupled with a Prussian blue cathode demonstrated a high capacity retention of 89.1% after 150 cycles at 50 mA g^−1^ and obtained great rate performance (Figure 8i). The primary advantage is the decoupling of interface design from electrolyte chemistry, allowing for the use of high‐performance but corrosive electrolytes (e.g., concentrated ethers) that would otherwise destroy a native SEI. The artificial HSEI acts as a universal protective cloak. Its design must address several requirements simultaneously: (1) high Na^+^ transference number to mitigate concentration polarization, (2) high mechanical modulus to resist dendrite intrusion, (3) excellent wettability with molten sodium for low interfacial resistance, and (4) electrochemical stability within the operating voltage window.

### 5.3 Solid‐State Electrolytes

The pursuit of solid‐state electrolytes (SSEs) can make safer and higher‐energy‐density metal‐sulfur batteries. Among various SSE design strategies, constructing heterostructure SSEs by integrating two or more phases with complementary properties at the nanoscale has emerged as a transformative approach to overcome the inherent limitations of single‐component systems. This design method moves beyond simple composites, aiming to create new ionic transport pathways and stabilized interfaces through synergistic interactions between the constituent phases. The following discussion outlines the unique advantages of heterostructure SSEs, the common strategies for their realization, and the consequent implications for sulfur cathode design.

**FIGURE 9:**
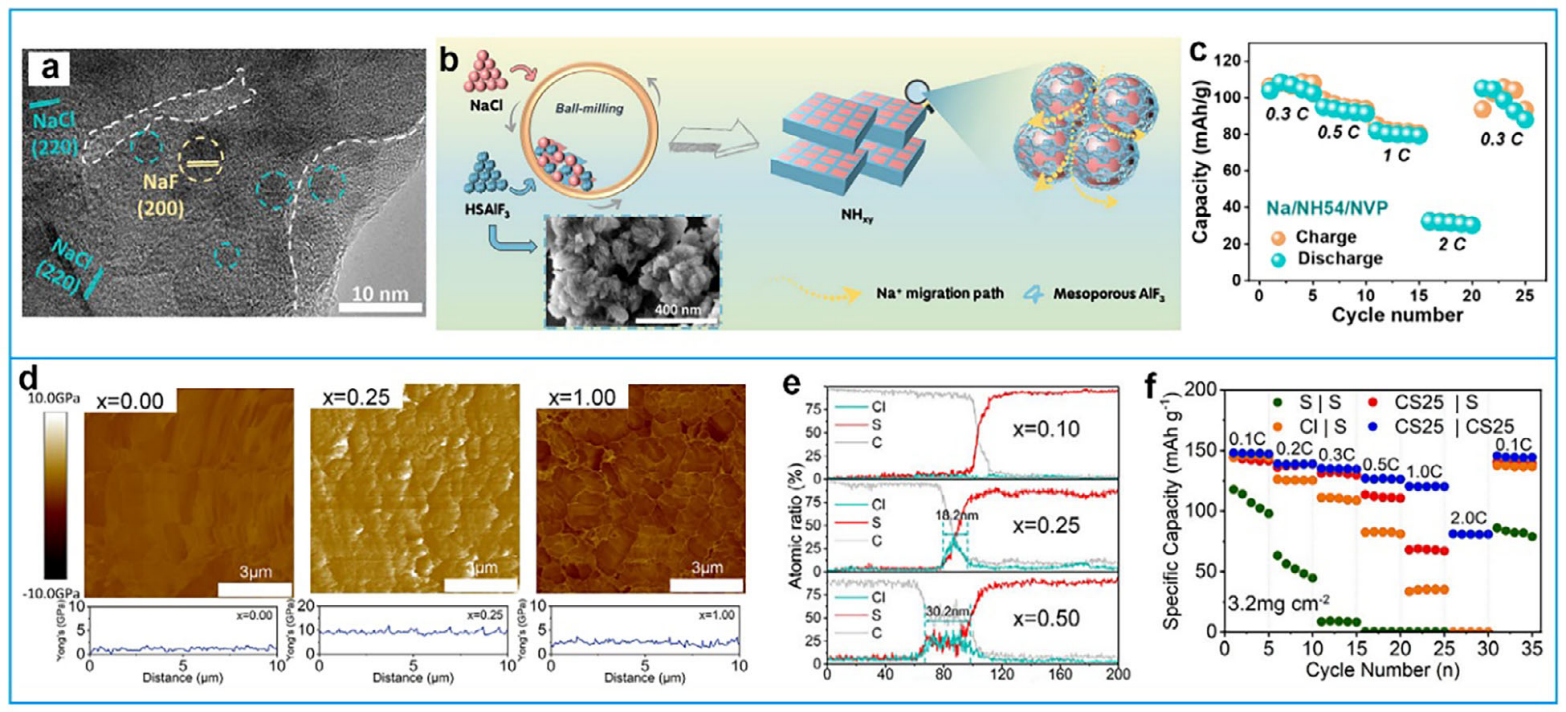
Advantages of heterostructured solid‐state electrolytes for RT Na‐S batteries. (a) HRTEM images of the NH54. (b) Schematic diagram of the synthesis process of NHxy from NaCl and HS‐AlF_3_, with an inset showing the SEM image of HS‐AlF_3_. (c) Rate the performance of Na/NH54/NVP composite. Reproduced with permission [170]. Copyright 2025, Wiley‐VCH. (d) AFM images of the SE pellets. (e) TEM‐EDS. (f) Rate capability of four different battery configurations measure. Reproduced with permission [170]. Copyright 2025, Wiley‐VCH.

Traditional SSEs, such as sulfides, oxides, and polymers, often face a performance trade‐off. Sulfides offer high ionic conductivity but suffer from narrow electrochemical windows and poor air stability, oxides are stable but exhibit high grain boundary resistance, and polymers are flexible but have low room‐temperature conductivity [171, 172, 173]. Heterostructure engineering provides a pathway to break this trade‐off by creating materials with synergistically enhanced properties that are unattainable by any single phase. Overall, the heterostructured SSEs exhibit several advantages. First, they can realize the dramatic enhancement of ionic conductivity, particularly at room temperature. The heterointerface itself can become a fast ion‐conduction highway. t Constructing nanoscaled heterostructures between phases like mesoporous AlF_3_ and NaCl can create rich defect regions and amorphous boundaries. These interfacial structures drastically lower the activation energy for ion migration, enabling ionic conductivities on the order of 10^−4^ S/cm at 30 °C, which is highly competitive for practical applications [174]. Similarly, introducing a secondary phase (e.g., a chloride‐based phase) to soften and bond the grain boundaries of a primary fluoride matrix has been shown to facilitate Na^+^ transport, significantly boosting conductivity. Second, heterostructure design can impart remarkable air stability. In one notable example, a fluoride‐chloride heterostructure electrolyte (NHxy) retained high conductivity even after exposure to 35% relative humidity, a feat attributed to the protective role of the stable fluoride phase (Figure 9a–c) [174]. This stability is crucial for scalable low‐cost manufacturing. Furthermore, integrating a soft phase (e.g., a polymer or a low‐melting‐point salt) with a rigid inorganic phase can improve the mechanical flexibility and interfacial contact of the electrolyte membrane, addressing the brittleness common in ceramic SSEs [175]. Third, by employing a core–shell structure where a high‐voltage‐stable phase (e.g., a halide) encapsulates a high‐conductivity phase (e.g., a sulfide), the resulting heterostructure electrolyte can achieve a wide voltage window (>4.0 V vs. Na^+^/Na) while maintaining good ionic transport. This design effectively isolates the sulfide from direct contact with the high‐voltage cathode, suppressing detrimental interfacial reactions and enabling the use of high‐energy‐density cathodes [176].

Generally, the fabrication of heterostructure SSEs requires precise control over phase distribution and interfacial intimacy. The first kind of mainstream fabrication strategy is based on the in situ reaction and phase boundary engineering. This involves triggering controlled chemical reactions between precursors to form in situ interconnected phases. For example, high‐energy ball milling of NaBr, NaCl, and mesoporous AlF_3_ can induce an interfacial anion exchange reaction, leading to a highly dispersed fluoride‐chloride‐bromide heterostructure with abundant conductive boundaries. The second kind of strategy is melt infiltration and grain boundary modification. It needs to infiltrate a low‐melting‐point ionic conductor (e.g., a chloride‐based eutectic melt) into the pores or grain boundaries of a rigid framework (e.g., a porous fluoride). The molten phase softens the rigid grain boundaries, fills voids, and creates continuous ion transport networks along the interfaces, significantly reducing grain boundary resistance. The third kind of strategy is the design of core–shell and laminate architectures. Such deliberate design involves fabricating particles or layers with a core–shell morphology. For example, creating a sulfide core with a thin uniform halide shell (NYZC‐NPS) effectively combines high conductivity with interfacial stability against high‐voltage cathodes (Figure 9d,e) [176]. This can greatly improve the electrochemical performance of full cells, especially their rate capacities (Figure 9f). Similarly, laminating different functional layers (e.g., a stiff ion‐conducting layer with a soft polymer layer) can create a mechanically reinforced, ultra‐lightweight composite electrolyte [175].

It needs to be specially pointed out that adopting heterostructured SSEs necessitates further sulfur cathode design. The goal should shift from merely confining polysulfides in a liquid electrolyte to establishing an efficient, stable, and mixed ion‐electron conduction network within an all‐solid composite. On the one hand, the sulfur cathode must transform into a finely structured composite, where the electronic conductor (e.g., carbon), the ion‐conducting heterostructure SSE, and the active sulfur (or Li_2_S/Na_2_S) are homogeneously mixed at the nanoscale. This can maximize the triple‐phase boundaries where redox reactions occur. A promising strategy is the in situ generation of a nanoscale mixed conductor within the cathode. On the other hand, heterostructure SSEs can be directly incorporated as an active filler within the cathodes. Beyond providing ion conduction, they can serve as built‐in catalysts and adsorbents. The polar surface of the SSE attracts polysulfides and provides alternative Na ion transport pathways, mitigating the viscosity and ionic blockage issues in concentrated catholytes. This principle is directly transferable to all‐solid‐state systems using heterostructure SSE additives. Moreover, the volume change of the sulfur cathode during cycling poses a major challenge for solid‐state interfaces. The cathode architecture must accommodate this strain to maintain intimate contact with the SSEs. This can be achieved by designing a flexible and resilient cathode matrix. Using nano‐sized active materials minimizes absolute particle expansion and fracture. Additionally, incorporating a ductile component within the cathode composite (e.g., a small amount of polymer or soft carbon) can buffer the mechanical stress, ensuring cyclic stability of the rigid solid‐solid interface.

### 5.4 Current Collectors

The strategic applications of heterostructure engineering can also be extended to current collectors, which is the foundational components that distribute electrons and support the active layers. By constructing a functional heterostructured interface on the current collector surface, a proactive regime of electrochemical processes can be established to fundamentally address challenges at both electrodes. This discussion progresses from the regulation of sodium deposition at the anode to the stabilization of sulfur redox at the cathode, culminating in the specialized design for polysulfide catholyte configurations.

#### 5.4.1 Anode‐Side Current Collectors

For sodium metal anodes, the primary challenge is the chaotic nucleation and dendritic growth of sodium. Traditional planar foils (e.g., Cu and Al) are sodiophobic and uneven, leading to high nucleation overpotential and localized electric fields that induce dendrites. Heterostructure‐modified current collectors can address this issue by introducing sodiophilic and catalytic interfaces that dictate uniform deposition. The central principle involves the design of a surface with abundant and well‐defined active sites that can guide sodium‐ion flux. For instance, constructing a seamless Ni_3_S_2_/Ni_3_P heterostructure on 3D nickel foam (Ni_3_S_2_/Ni_3_P@NF) creates an advanced current collector [177]. The heterointerface provides numerous active sites for uniform Na^+^ deposition, and more importantly, its high work function effectively lowers the local Fermi level. This modulation suppresses continuous electron tunneling to the electrolyte, thereby stabilizing the SEI and mitigating parasitic decomposition, in favor of long‐term cyclability.

**FIGURE 10:**
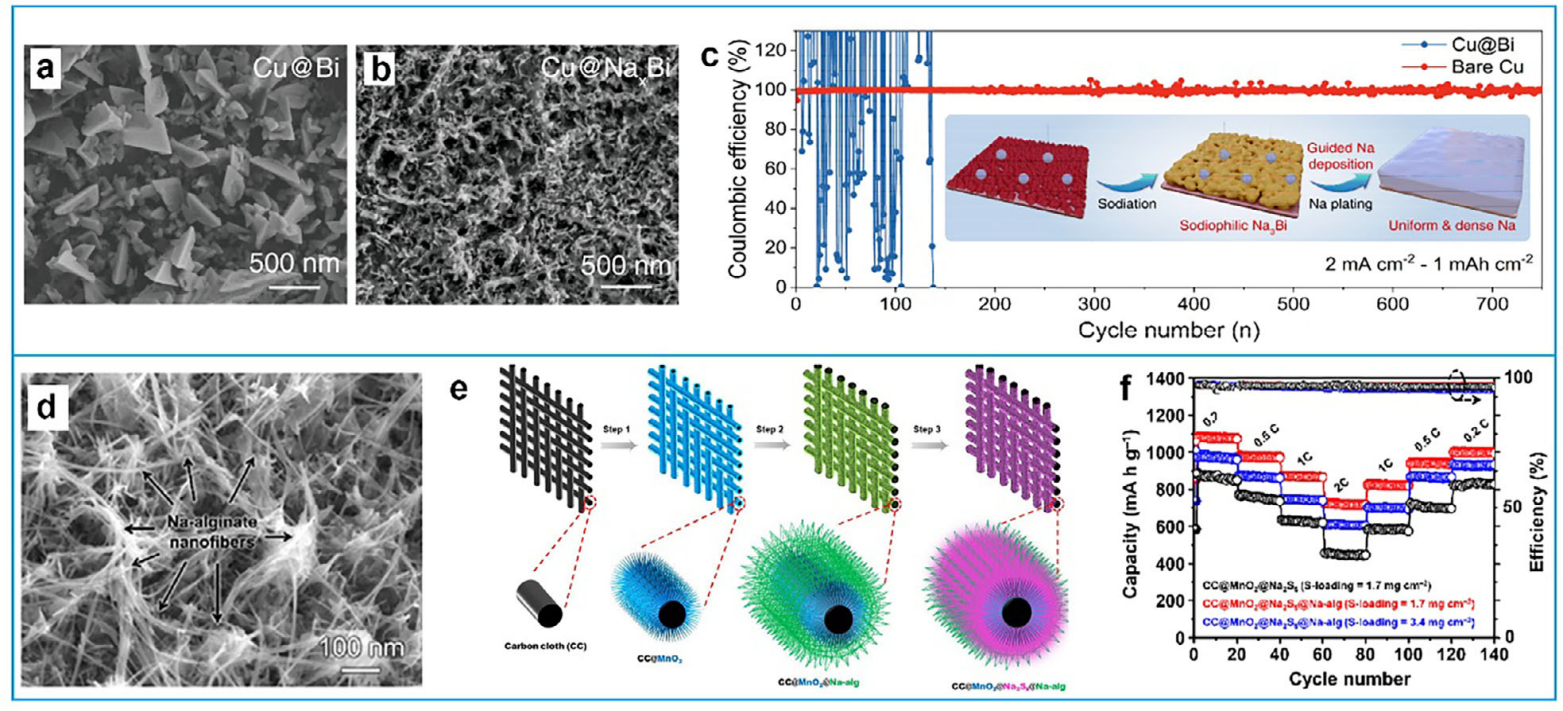
Heterostructure design for current collectors in RT Na‐S batteries. (a) SEM image of the Cu@Bi. (b) SEM image of the Cu@Na_x_Bi. (c) CE of Na plating/stripping at 2 mA cm^−2^ and 1 mA h cm^−2^ for the Cu@Bi||Na and the Bare Cu||Na. Reproduced with permission [170]. Copyright 2023, MDPI. (d) SEM images of the CC@MnO_2_@Na‐alg substrate. (e) Schematic illustration of CC@MnO_2_@Na_2_S_6_@Na‐alg cathode preparation. (f) Rate performance of the CC@MnO_2_@Na_2_S_6_ cathode and CC@MnO_2_@Na_2_S_6_@Na‐alg cathodes at 1C. Reproduced with permission [184]. Copyright 2021, American Chemistry Society.

An alternative strategy leverages in situ alloying reactions to form a sodiophilic layer. For instance, a simple galvanic replacement reaction can create a bismuth nanoparticle coating on copper foil (Cu@Bi) [178]. Figure 10a,b show the microscopic morphologies of the as‐designed Cu@Bi. Upon initial contact with sodium, the Bi transforms into a porous Na_3_Bi alloy phase. Such a porous alloy structure offers high sodium affinity, homogenizes ion flux, reduces nucleation overpotential, and finally guides the growth of a dense sodium metal layer. Thus, the sodium metal batteries realized high Coulombic efficiency during the long cycling process (Figure 10c).

#### 5.4.2 Cathode‐Side Current Collectors

The sulfur cathode presents a different set of demands: corrosion resistance against polysulfides, efficient electron supply to insulating sulfur species, and in some designs, a degree of polysulfide confinement. Heterostructure design for cathode‐side current collectors need to focus on compatibility, conductivity, and catalysis

A robust, conductive, and chemically stable first layer is essential to sulfur cathodes. Modern heterostructure designs build upon this by integrating catalytic functionality. A current collector coated with a heterostructure of a conductive phase (e.g., metal nitride) and a catalytic phase (e.g., metal oxide) can serve a dual purpose. It ensures rapid electron transport while actively catalyzing the conversion of nearby polysulfides, effectively functioning as an extension of the sulfur host's catalytic activity directly at the current collector interface.

#### 5.4.3 Design Considerations for Polysulfide Catholyte

The polysulfide catholyte configuration, where sulfur is pre‐dissolved as polysulfides in the electrolyte, represents a distinct and promising cell design, aiming at achieving high sulfur loading and utilization. This system bypasses the solid S_8_ dissolution step but places unique demands on the current collector as it is in constant contact with a highly concentrated, reactive, and mobile polysulfide solution. In this architecture, the current collector must transcend its traditional role and function as a multifunctional substrate that integrates adsorption, catalysis, and conduction. In this context, the current collectors must collect current as well as actively anchor and convert the dissolved active materials directly on its surface. The heterostructure design, therefore, needs to maximize the density of polar and catalytic sites. To this end, the design of heterostructured current collectors have to follow two key principles: (1) material selection and surface engineering. Carbon‐based materials like carbon paper are often preferred over aluminum due to their better chemical stability and lower catalytic activity toward unwanted side reactions. The surface should be engineered into a porous, high‐surface‐area 3D scaffold (e.g., carbon felt, foam) to increase reactive interface area. (2) heterostructure functionality. A conformal coating of a tailored heterostructure (e.g., Mo_2_N–W_2_N, transition metal sulfide/phosphide) on this 3D scaffold is critical.

Generally speaking, an ideal heterostructure should provide: (1) strong chemisorption to localize the dissolved polysulfides near the electron source, preventing their diffusion away and subsequent loss of active material [179]; (2) high catalytic activity to rapidly catalyze the redox reactions between different polysulfide chain lengths (S_n_^2−^ ↔ S^2−^), ensuring fast reaction kinetics despite the absence of a traditional sulfur host [180]; (3) unobstructed porosity to allow for electrolyte wetting and ion transport while providing ample space for potential solid Na_2_S_2_/Na_2_S deposition during discharge.

Recently, Sagar Mitra et al., proposed a series of current collectors with heterostructure features for catholyte in RT Na‐S batteries, involving activated carbon cloth [181], the ACC decorated with indium tin oxide nanoparticles (ITO@ACC), [182] MnO_2_ nanoarrays on carbon cloth (CC@MnO_2_) [183], and MnO_2_ nanoarray‐decorated carbon cloth with sodium alginate nanofibers (CC@MnO_2_@Na‐alg) [184] et. For example, in the case of CC@MnO_2_@Na‐alg, the alginate not only provided strong adhesion but also anchored polysulfides via oxygen‐containing groups. The cathode with 3.4 mg cm^−2^ sulfur loading delivered 752 mA h g^−1^ at 1C with 94% retention after 1000 cycles. In another design of CC@MnO_2_, the in situ formed polythionate complexes chemically bonded with polysulfides and mediated their redox reactions, enabling an energy density of 946 W h kg^−1^ and a capacity of 938 mA h g^−1^ at 0.2 A g^−1^ with 67% retention after 500 cycles.

## 6 Conclusion and Perspectives

### 6.1 Summary

This review has systematically deconstructed the roles of heterostructure materials in RT Na‐S batteries, presenting them not as a mere material choice but as a fundamental design methodology. We have demonstrated that the strategic creation of interfaces between dissimilar phases, encompassing carbon‐carbon, carbon‐transition metal compound, single‐atom catalysts, and all‐inorganic composites, etc., confers a universal set of advantages in strong chemisorption, enhanced catalytic activity, optimized charge transport, and improved mechanical stability, etc. These advantages are ingeniously deployed across the battery architectures, transforming every component from a passive entity into an active regulator of electrochemical reaction processes. The heterostructure engineering enables precise interfacial microenvironments and offers a coherent multi‐front strategy to combat the fundamental challenges of the RT Na‐S chemistry.

### 6.2 Perspectives

**FIGURE 11:**
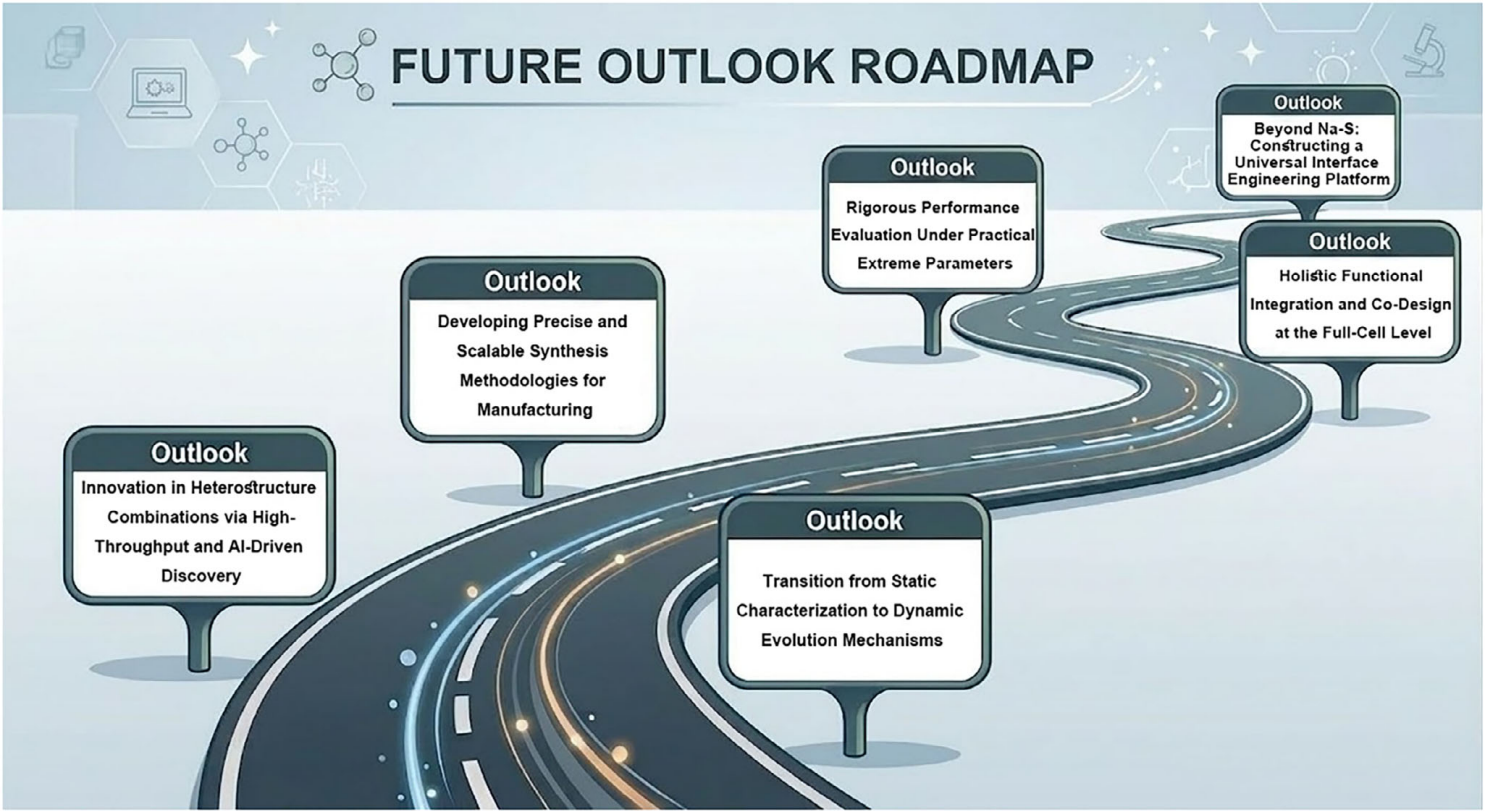
Perspectives of heterostructured materials for RT Na‐S batteries.

While significant progress has been made, the road from a compelling concept to a commercially viable technology demands a concerted effort to address deeper scientific questions and practical hurdles. Herein, we propose some targeted and interconnected perspectives to guide future research toward a new level of sophistication and application relevance (Figure 11).

1. Future research must transition from analyzing static pristine interfaces to probing their dynamic evolution under operating conditions. This requires advanced in situ/operando techniques to track real‐time morphological, compositional, and electronic state changes during cycling. Key questions include the mechanism by which the built‐in electric field redistributes upon polysulfide adsorption, the behavior patterns that the constituent phases undergo reconstruction or elemental interdiffusion, and a deep understanding of methods to realize durable interface layers that are not only initially active but also dynamically stable or self‐healing over thousands of cycles.
2. The exploration of material pairs must evolve beyond empirical trials. A promising frontier employs high‐throughput computational screening and artificial intelligence to discover novel combinations. Machine learning models trained on existing data can predict the formation energy and catalytic activity of thousands of potential heterostructures, guiding the synthesis of unexplored families, such as junctions between two‐dimensional materials and high‐entropy compounds, to unlock unprecedented functionalities.
3. Translating lab‐scale syntheses into viable technologies requires robust and scalable methods. Future research should focus on techniques that offer inherent control, such as atomic layer deposition for conformal core–shell structures or flow chemistry for continuous nanocrystal production, while maintaining exquisite interfacial control at a kilogram‐scale relevant to manufacturing. Bridging nanoscience and battery production is a critical challenge.
4. Future work must transcend component‐level optimization and embrace full‐cell engineering. The heterostructured sulfur host, anode protector, separator coating, and current collector must be designed as an integrated system. This involves matching kinetics, ensuring chemical compatibility, and managing mechanical stack pressure. The performance of a perfect cathode can be nullified by a poorly matched anode interface, making system‐level synergy the ultimate goal.
5. The true merit of any design must be validated under conditions mirroring commercial requirements. A rigorous reporting standard is mandated, including: high sulfur loading (>7 mg cm^−2^), lean electrolyte (E/S ratio < 3 µL mg^−1^), low N/P ratio (< 3), and testing in pouch cell formats. Research must address how heterostructures fail under these constraints, for example, pore clogging, catalytic poisoning, and mechanical delamination, etc.
6. The principles of interface engineering elucidated constitute a universal framework for electrochemical energy storage. This knowledge should be actively adapted to confront analogous challenges in other beyond‐lithium/sodium systems, such as other metal‐sulfur (K─S, Al─S, Ca─S, Zn─S, and Mg─S) batteries, conversion‐type cathodes, and even electrocatalytic systems for reactions like CO_2_ reduction. The insights gained from tailoring interfaces for polysulfide adsorption and sodium nucleation can catalyze advances across a broad frontier of energy science.
7. The outstanding performance of heterostructure materials demonstrated in coin cells urgently requires re‐validation under pouch cell conditions characterized by high sulfur loading (≥5 mg cm^−2^), lean electrolytes, and thin sodium anodes. Future endeavors should focus on (1) developing scalable fabrication technologies to ensure the mechanical integrity and catalytic activity of heterogeneous interfaces within thick electrodes; systematically evaluating failure modes under lean‐electrolyte conditions; and (2) establishing standardized pouch cell testing protocols to propel heterostructure materials from laboratory research to practical device applications.

In conclusion, heterostructure engineering has provided a powerful lens through which to re‐imagine the RT Na‐S batteries. The path forward demands a deeper understanding of dynamic interfaces, innovation in their scalable and intelligent creation, and rigorous validation within integrated practical systems. By embracing these forward‐looking perspectives, the scientific community can solidify the role of heterostructures as a cornerstone for next‐generation energy storage.

## Author Contributions

**Yeqing Yang**: conceptualization, validation, investigation, writing – original draft, project administration, resources. **Xue Li**: conceptualization, methodology, formal analysis, data curation, visualization, writing – original draft. **Xiang‐Long Huang**: writing – original draft, writing – review and editing, funding acquisition, supervision. **Kunjie Zhu**: writing – review and editing, funding acquisition, software. **Yun‐Xiao Wang**: writing – review and editing, supervision, funding acquisition, resources.

## Conflicts of Interest

The authors declare no conflicts of interest.

## Data Availability

The data that support the findings of this study are available on request from the corresponding author. The data are not publicly available due to privacy or ethical restrictions.
